# Arabidopsis GCN2 kinase contributes to ABA homeostasis and stomatal immunity

**DOI:** 10.1038/s42003-019-0544-x

**Published:** 2019-08-08

**Authors:** Xiaoyu Liu, Taiaba Afrin, Karolina M. Pajerowska-Mukhtar

**Affiliations:** 1 Department of Biology, University of Alabama at Birmingham, 1300 University Blvd., Birmingham, AL 35294 USA; 2 Present Address: Bayer Crop Science, 800 N Lindbergh Blvd., Creve Coeur, MO 63144 USA

**Keywords:** Biotic, Stomata

## Abstract

General Control Non-derepressible 2 (GCN2) is an evolutionarily conserved serine/threonine kinase that modulates amino acid homeostasis in response to nutrient deprivation in yeast, human and other eukaryotes. However, the GCN2 signaling pathway in plants remains largely unknown. Here, we demonstrate that in Arabidopsis, bacterial infection activates AtGCN2-mediated phosphorylation of eIF2α and promotes TBF1 translational derepression. Consequently, TBF1 regulates a subset of abscisic acid signaling components to modulate pre-invasive immunity. We show that GCN2 fine-tunes abscisic acid accumulation and signaling during both pre-invasive and post-invasive stages of an infection event. Finally, we also demonstrate that AtGCN2 participates in signaling triggered by phytotoxin coronatine secreted by *P*. *syringae*. During the preinvasive phase, AtGCN2 regulates stomatal immunity by affecting pathogen-triggered stomatal closure and coronatine-mediated stomatal reopening. Our conclusions support a conserved role of GCN2 in various forms of immune responses across kingdoms, highlighting GCN2’s importance in studies on both plant and mammalian immunology.

## Abstract (web summary)

Xiaoyu Liu et al. show that Arabidopsis GCN2 kinase plays a role in abscisic acid homeostasis and stomatal immunity by regulating stomatal closure and reopening in response to pathogenic bacteria or their toxin, respectively. This study suggests an evolutionarily conserved role for GCN2 in plant immune response.

## Introduction

Cellular robustness and resilience, universal features of all biological systems, allow organisms to withstand internal and environmental perturbations^1,2^. Cells respond to these changes by trading off growth-related processes with stress-associated signaling cascades that are manifested by distinctive regulatory programs^3,4^. These responses require the activation of specific master regulator(s) to maintain cellular homeostasis^5^. General control non-derepressible 2 (GCN2), a universal regulator, is an evolutionarily conserved sensor involved in perception of nutrient starvation, stress signal transduction cascades, and diverse immune responses^5,6^. *GCN2* encodes a protein kinase with a conserved N-terminal kinase domain and a C-terminal region homologous to histidyl-tRNA synthetase (HisRS)^7^. In yeast and mammals, GCN2 senses amino acid starvation by binding with the uncharged tRNAs via its HisRS domain^8,9^. This in turn stimulates the kinase activity of GCN2 and initiates a downstream signaling cascade^10,11^. In particular, GCN2 phosphorylates eukaryotic initiation factor alpha (eIF2α), causing its reduced mRNA scanning capacity, which leads to an arrest of general translation but initiates translation of the main GCN4 ORF (mORF)^11,12^. The GCN2/eIF2α-mediated translation switch of GCN4 activates downstream target genes and alleviates the starvation stress in yeast. Likewise, mammals also possess a functional eIF2α phosphorylation switch that can be activated by four diverse kinases including GCN2^13^. Upon the perception of stress caused by essential amino acid starvation, eIF2α phosphorylation allows for uORF-mediated translation of ATF4 transcription factor (TF). Arabidopsis contains a single copy of GCN2 (At3g59410) that was shown to functionally complement *Δgcn2* yeast mutant strain^7^. Similar to the mammalian and yeast systems, AtGCN2 can respond to branched amino acid deprivation and diverse abiotic stress conditions, including heat and osmotic stress, treatments with herbicides, phytohormones, as well as wounding^14–18^, suggesting the functional conservation of these molecules across kingdoms. AtGCN2 stress signaling was also demonstrated to be inducible by β-aminobutyric acid (BABA)^19,20^. BABA-induced priming is associated with eIF2α phosphorylation, which was absent in the *atgcn2* mutant plants^19^. The *atgcn2* mutants are more tolerant to BABA-induced growth repression, but display normal BABA-induced resistance against *Hyaloperonospora arabidopsidis* isolate Cala2, which is virulent on the L*er* accession^19^.

Previously, we showed the roles of AtGCN2 in gibberellic acid (GA)-mediated plant and seed development^21^. Moreover, we identified a heat-shock factor-like TF, TBF1 (HSF4/HsfB1) that contains uORFs in its 5′ UTR, reminiscent of the mammalian ATF4 and yeast GCN4^3,22,23^. We also demonstrated that TBF1 is translationally regulated through uORF-mediated translation derepression upon pathogen infection^3^. This body of evidence collectively suggests possible existence of a GCN2-mediated signaling cascade in Arabidopsis that could play important roles in plant growth and development, as well as immune responses^21,24^.

The plant immune system utilizes an array of phytohormones to coordinate the defense response. The fine-tuned signaling interplay among salicylic acid (SA), jasmonic acid (JA), ethylene, abscisic acid, (ABA), GA, and brassinosteroids^4,25,26^ facilitates an integrated defense response^4,27^ that often relies on antagonistic hormone action^28^. For instance, over-accumulation of SA inversely affects JA levels and these events positively contribute to the establishment of immunity against bacterium *Pseudomonas syringae* pv. *tomato* DC3000 (*Pst* DC3000)^29,30^. Virulent pathogens manipulate this hormonal crosstalk to diminish immune responses and thus drive the cell toward susceptibility. For example, *Pst* DC3000 delivers virulence factors (hereafter effectors) through the type-III secretion system (TTSS) into the host cell^31,32^. *Pst* DC3000 *hrcC* strain, a mutant defective in TTSS, is almost nonpathogenic^33^. In addition to the effectors, *Pst* DC3000 also delivers toxin coronatine (COR), a structural mimic of JA-isoleucine (an active form of JA) to antagonize SA-mediated defenses and cause plant disease^34–36^.

Emerging roles of ABA during different phases of bacterial infection have also been described^34,37^. A positive role for ABA in defense during early (preinvasive) stages of bacterial infection is supported by its well-known function in the regulation of ion channels flux, facilitating the closure of stomata. On the contrary, ABA can suppress defense responses through its antagonistic interaction with SA and possibly other hormones, in the later (postinvasive) phase of infection^37^.

We previously reported that AtGCN2 negatively regulates defense responses against phytopathogens with diverse lifestyles^24^. Here, we set out to study the pathogen infection-triggered AtGCN2-mediated signaling. We demonstrated that bacterial infection initiates AtGCN2-dependent eIF2α phosphorylation. Moreover, our genetic data indicate that AtGCN2 participates in uORF-mediated translational derepression of TBF1 and executes dual roles in stomatal immunity by contributing to pathogen-triggered ABA-dependent stomatal closure and to COR-mediated stomatal reopening. Conversely, however, AtGCN2 acts as a negative regulator of plant immunity during the postinvasive stage of infection, mainly by regulating ABA accumulation and TBF1-dependent transcription of ABA signaling components. In summary, we uncovered opposing roles of AtGCN2 in regulating different layers of plant immunity.

## Results

### Pathogen triggers AtGCN2-dependent eIF2α phosphorylation

**Fig. 1:**
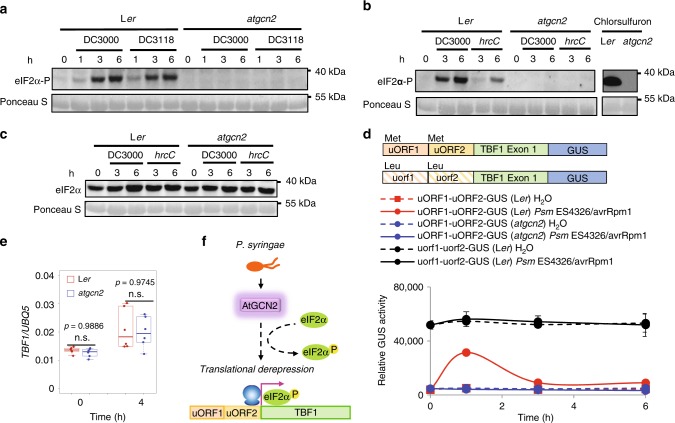
AtGCN2 is required for *P*. *syringae*-trigged eIF2α phosphorylation and TBF1 translational derepression. **a** Detection of phosphorylated form of eIF2α in the samples prepared from 2-week-old plants treated with *Pst* DC3000 or *Pst* DC3118 (OD_600 nm_ = 0.02). Phosphorylation state-specific (S51) antihuman eIF2α antibody was used. Ponceau S staining shows loading amounts. Full blots are shown in Supplementary Fig. 1b. **b** Detection of phosphorylated form of eIF2α in the samples prepared from 2-week-old plants treated with *Pst* DC3000 or *Pst* hrcC (OD_600 nm_ = 0.02). Phosphorylation state-specific (S51) antihuman eIF2α antibody was used. Ponceau S staining shows loading amounts. Full blots are shown in Supplementary Fig. 1d. **c** Time course total eIF2α protein accumulation in 2-week-old plants upon *Pst* DC3000 or *Pst* hrcC (OD_600 nm_ = 0.02) challenge. Ponceau S staining shows loading amounts. Full blots are shown in Supplementary Fig. 1h. **d** Schematic diagram of constructs used for quantification of GUS activity. Start codon and mutated start codon are labeled as Met and Leu, respectively. GUS activity is shown in T_3_ plants uORF1-uORF2-GUS (L*er*), uorf1-uorf2-GUS (L*er*), and uORF1-uORF2-GUS (*atgcn2*) at designated time points after inoculation with *Psm* ES4326/avrRpm1 or control. Error bars represent standard deviation of three technical replicates. Experiments were conducted in three independent biological replications with similar results. **e** Transcript accumulation of *TBF1* was measured in 4-week-old L*er* and *atgcn2* plants that were sprayed with salicylic acid (SA; 0.5 mM) using real-time RT-PCR. The boxes plots extend from the 25th to 75th percentiles and the whiskers extend from the minimum to the maximum level. Median values are plotted in the boxes with data generated from three independent biological replicates. Statistical analysis was performed with two-way ANOVA with Tukey’s test (significance set at *P* ≤ 0.05) and n.s. denotes not significant. **f** Schematic representation of AtGCN2- and TBF1-mediated signaling events following *P*. *syringae* infection. AtGCN2 phosphorylates eIF2α following *P*. *syringae* infection and might be directly or indirectly involved in translational regulation of TBF1

Activation of the GCN2/eIF2α signaling and uORF-mediated translational derepression of a master TF(s) has been described in mammals and yeast^10,11^. To determine the existence of such a pathway in Arabidopsis in response to a bacterial infection, we first analyzed the eIF2α phosphorylation status in the wild-type L*er* and *atgcn2* loss-of-function mutant that is completely deficient in AtGCN2 transcription (Supplementary Fig. 1a). We used a phosphorylation state-specific antihuman eIF2α antibody, which can specifically recognize phosphorylated eIF2α in Arabidopsis in response to various abiotic stressors, including starvation, UV irradiation, wounding, cold temperature, NaCl, and H_2_O_2_^3,15,18^. We detected eIF2α phosphorylation in the wild-type plants as early as 1 h post inoculation (hpi) with the virulent bacterium *Pst* DC3000 and mutant COR-deficient strain *Pst* DC3118^38^ (Fig. 1a and Supplementary Fig. 1b). As expected, the control treatment did not induce eIF2α phosphorylation, confirming its specificity to pathogen challenge (Supplementary Fig. 1c, d). This phosphorylated form of eIF2α continued to accumulate at 3 hpi and 6 hpi but was absent in the *atgcn2* mutant plants at all the tested time points, suggesting the requirement of AtGCN2 in eIF2α phosphorylation during plant infection (Fig. 1a and Supplementary Fig. 1c, d). Subsequently, we tested if virulent effectors contribute to eIF2α phosphorylation by comparing *Pst* DC3000 and *Pst* DC3000 *hrcC* strain that is defective in TTSS-mediated effector delivery. *Pst hrcC* induced a much weaker eIF2α phosphorylation than DC3000, indicating that the effector proteins or type-III pilus itself are required for the full extent of eIF2α phosphorylation (Fig. 1b and Supplementary Fig. 1e). Chlorsulfuron-treated plants were used as a positive control as this herbicide was previously shown to act as a powerful trigger of eIF2α phosphorylation^15^. Moreover, AtGCN2-dependent eIF2α phosphorylation can also be induced by *P. syringae* pv. *maculicola* ES4326 (*Psm* ES4326) expressing avrRpm1, confirming that AtGCN2 universally responds to stress caused by *P*. *syringae* infection irrespective of the strain (Supplementary Fig. 1f, g). The levels of transcript and total eIF2α protein in wild-type and *atgcn2* plants were comparable (Fig. 1c and Supplementary Fig. 1h–k), further confirming that the observed response is dynamic and phosphorylation mediated. Overall, these data suggest that AtGCN2 is required for the eIF2α phosphorylation following microbial infection.

### TBF1 translational derepression is AtGCN2-dependent

The high degree of structural and functional conservation of GCN2/eIF2α in Arabidopsis, yeast and human prompted us to investigate their downstream uORF-mediated targets^10,11,39–41^. Among others, the Arabidopsis TF TBF1 was proposed to be a possible target given that its mRNA contains two uORFs upstream of the main ORF (mORF) (Fig. 1d) and was shown to be translationally regulated^3^. To test the requirement of AtGCN2 in TBF1 translational derepression, we employed reporter constructs by fusing in-frame either wild-type uORFs or uorf (mutated form of uORF harboring a mutation in the initiator codon, i.e., ATG to CTG) and the first exon of mORF TBF1 to GUS reporter coding sequences as previously described^3^. The resulting constructs were transformed into L*er* and *atgcn2* plants to generate stable transgenic lines. We challenged L*er* plants expressing uORF1-uORF2-GUS or uorf1-uorf2-GUS as well as *atgcn2* expressing uORF1-uORF2-GUS with an avirulent bacterial pathogen and quantified GUS activity. As shown in Fig. 1d, we detected an eightfold increase in the GUS activity in the uORF1-uORF2-GUS (in L*er* background) plants, while this induction was completely abolished in the uorf1-uorf2-GUS (in L*er* background) and uORF1-uORF2-GUS (in *atgcn2* background) plants. The uorf1-uorf2-GUS (in L*er* background) plants lost their translational-level inhibition and displayed a high output of basal level translation, which is not further inducible, since the uORF cassette is mutated and not reactive to stimulation (Fig. 1d). Given that the *atgcn2* mutant displays no defects in accumulating TBF1 transcript (Fig. 1e), and comparable levels of *uidA-TBF1* chimeric transcript expression were detected in the three transgenic reporter lines following pathogen challenge (Supplementary Fig. 1i, j), the measured GUS activity represents the rate of translation reinitiation at the TBF1 mORF. Taken together, our data indicate that Arabidopsis GCN2 is implicated in translational regulation of TBF1 in plant immune signaling (Fig. 1f).

### AtGCN2 and TBF1 regulate preinvasive stage ABA signaling

**Fig. 2:**
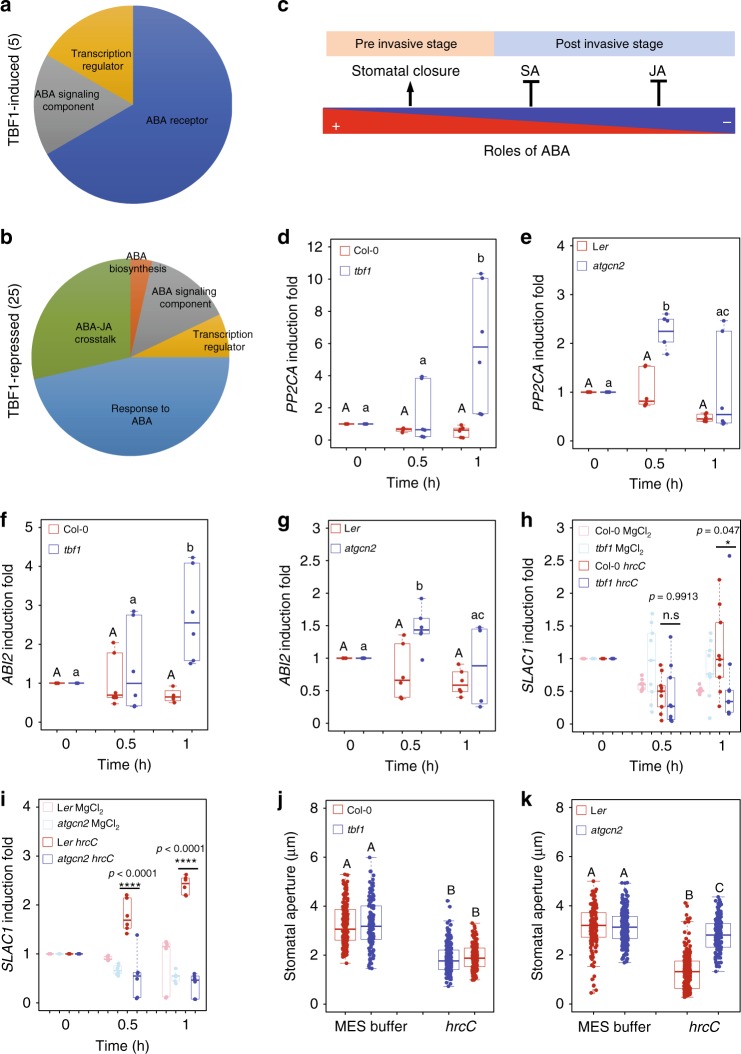
AtGCN2-TBF1 cascades transcriptionally manipulate ABA signaling components during preinvasive stage. Pie charts of GO terms of genes that are transcriptionally induced (**a**) and repressed (**b**) by TBF1 upon elf18 treatment. Details are listed in Supplementary Table 1. **c** Diagrammatic representation of a model illustrating the opposing roles of ABA in preinvasive and postinvasive phases of pathogen infection. Positive and negative contributions of ABA in plant defense are depicted in blue and red colors, respectively. Salicylic acid (SA) and jasmonic acid (JA) are shown [adopted from Ton et al.^37^]. Real-time RT-PCR analyses were performed on leaf samples of plants spray inoculated with *Pst* hrcC (OD_600nm_ = 0.2) and transcript levels of *PP2CA* (**d**, **e**), *ABI2* (**f**, **g**), and *SLAC1* (**h**, **i**), respectively, were quantified. The box plots are prepared as described above. Median values are plotted in the boxes with data generated from three independent biological replicates. For **d**–**g**, two-way ANOVA with Tukey’s test (significance set at *p* ≤ 0.05) was performed; capital letters denote difference in wild-type plants (L*er* or Col-0), and lower case letters denote difference in mutant plants (*tbf1* or *atcgn2*). For **h**, **i**, two-way ANOVA with Tukey’s test was performed and asterisks above the bars signify statistically significant differences between *Pst* hrcC challenged Col-0 and *tbf1* or L*er* and *atgcn2* plants (**p ≤ 0.01, ***p ≤ 0.001, n.s.—not significant). **j**, **k** Stomatal aperture width was measured in epidermal peels of 4-week-old *tbf1* mutants (in Col-0 background; **j**) and *atgcn2* mutants (in L*er* background; **k**) that were treated with *Pst hrcC* (OD_600nm_ = 0.2) for 1 h. The box plots are prepared as described above. Median values are plotted in the boxes with data generated from stomata derived from three independent biological replicates. One-way ANOVA with Tukey’s test (significance set at *p* ≤ 0.05) was performed and letters above the bars signify statistically significant differences among groups

To elucidate candidate cellular processes downstream of AtGCN2-dependent eIF2α phosphorylation, we conducted bioinformatics analyses of the TBF1-dependent immune transcriptome^3^. We discovered an enrichment of differentially expressed genes encoding ABA receptors, ABA biosynthetic enzymes and ABA-related transcriptional regulators (Fig. 2a, b and detailed information in Supplementary Table 1). We classified these genes into two categories, TBF1-induced and TBF1-repressed. Given that ABA was postulated to play dual roles during different stages of plant infection^37^, we proposed that the AtGCN2/eIF2α-mediated and TBF1-dependent signaling may differentially contribute to preinvasion- and postinvasion-associated defenses (Fig. 2c). Among the ABA-related TBF1-repressed genes, we selected two canonical negative ABA response regulators, *PP2CA* (*Protein Phosphatase type 2C*) and *ABI2* (*Abscisic Acid Insensitive 2* that also encodes a PP2C) for expression profiling analyses. Promoter sequence analyses of *PP2CA* and *ABI2* confirmed the presence of conserved *TL1* elements (*GAAGAAGAA*, the binding site for TBF1), in their upstream regulatory regions^3^ (Supplementary Table 1). This indicates that *ABI2 and PP2CA* may be transcriptionally regulated by TBF1. To experimentally support this observation, we carried out a real-time qPCR and analyzed expression profiles of these two genes in response to microbial challenge using the previously published loss-of-function *tbf1* mutant (Col-0 background)^3^. We observed transcriptional repression of *PP2CA* and *ABI2* in the Col-0 plants during the preinvasive stage of *Pst hrcC* infection; this suppression, however, was not detected in the *tbf1* mutant (Fig. 2d, f). Thus, this repression likely constitutes a general, and not effector-specific, regulatory effect of TBF1 on the ABA pathway in response to bacterial infection.

To examine the roles of AtGCN2 in the preinvasive phase of plant immunity, we monitored the temporal expression patterns of these ABA-responsive genes in the *atgcn2* plants in response to *Pst hrcC*. We observed a repression of *PP2CA* and *ABI2* mRNA levels in the wild-type infected with *Pst hrcC*, while the transcripts of these two ABA-related genes were elevated in the *atgcn2* mutant (Fig. 2e, g). The resemblance of *PP2CA* and *ABI2* transcriptional trends in *tbf1* and *atgcn2* plants indicates that TBF1 might function as one of the AtGCN2 targets to regulate the ABA response during the preinvasive stage of pathogen challenge.

Besides regulating TBF1 translational derepression, AtGCN2-mediated eIF2α phosphorylation leads to general attenuation of protein synthesis^15^. In yeast, GCN2 activation requires functional interactors Gcn1 and Gcn20^42–44^. Recently, Arabidopsis GCN1 and GCN20 were reported to share sequence similarity with yeast Gcn1 and Gcn20 and shown to play an important role in regulating stomatal immunity^45–47^. Moreover, another recent report illuminated the function of Arabidopsis GCN4, an AAA^+^-ATPase family protein, as a key regulator of stomatal aperture during stress^48^. Stomata, which serve as gateways for gas exchange, respond to environmental cues by changes in osmotic pressure within guard cells, allowing fine-tune regulation of the stomatal aperture^49^. It has been shown that treatments with immune stimuli result in a rapid stomata closure to restrict pathogen entrance^50^. In general, stomatal closure is executed through an efflux of ions through ion channels. Among them, an S-type anion channel SLAC1 (SLOW ANION CHANNEL-ASSOCIATED 1) plays a critical role in the pathogen-induced stomatal closure^50^. Quantification of *SLAC1* transcripts upon *Pst hrcC* challenge revealed that both the *tbf1* and *atgcn2* mutants are impaired in this response compared with their respective wild types (Fig. 2h, i), further implying a possible regulatory relationship between these two factors. Finally, we tested the *atgcn2* and *tbf1* mutants for their ability to execute stomatal closure in response to *Pst hrcC*. Our results demonstrated that the *atgcn2* mutant is less effective in closing the stomata following *Pst hrcC* infection (Fig. 2k), while *tbf1* plants show near wild-type levels of stomatal closure (Fig. 2j). This less severe stomatal phenotype of *tbf1* plants is consistent with the fact that TBF1 constitutes only one of many potential downstream targets of AtGCN2, and suggests that other factors might be able to compensate this response in the *tbf1* plants. Overall, our results demonstrate that AtGCN2 contributes to the regulation of ABA signaling and stomatal immunity during the preinvasive stage of bacterial infection. Moreover, AtGCN2 might have a regulatory relationship with the key immune TF TBF1 during the preinvasive infection stage.

### AtGCN2 is required for COR-mediated stomatal reopening

**Fig. 3:**
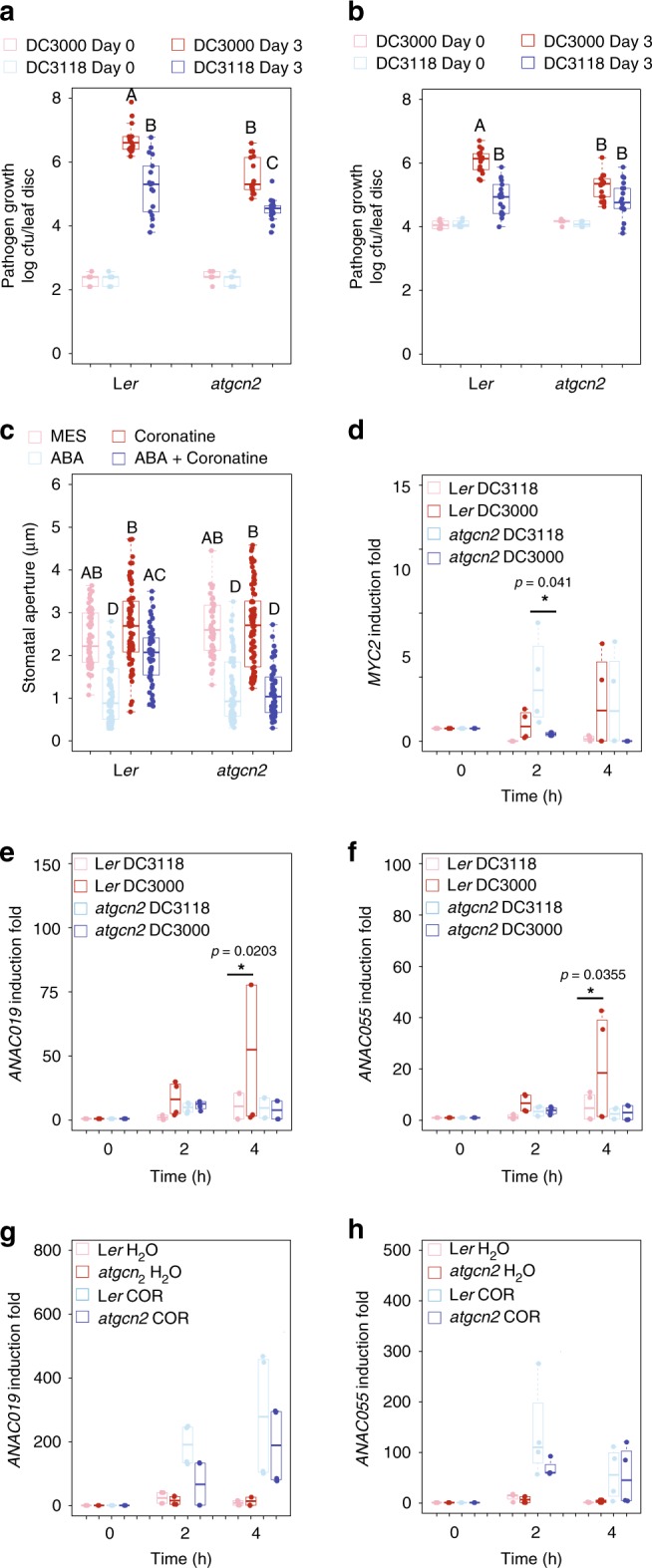
AtGCN2 contributes to stomatal immunity and affects disease susceptibility at the preinvasive stage of the infection event. Pathogen growth was quantified in 4-week-old plants infected with *Pst* DC3000 or *Pst* DC3118 at 3 days using syringe inoculation (OD_600 nm_ = 0.0002) (**a**) and spray inoculation (OD_600 nm_ = 0.2) (**b**). The box plots are prepared as described above. Median values are plotted in the boxes with data generated from three independent biological replicates with two different technical replicates of each biological replicate (day 0) or three independent biological replicates with six technical replicates of each biological replicate (day 3). Statistical analysis was performed by two-way ANOVA with Tukey’s test, letters above the bars signify statistically significant differences among groups (*p* ≤ 0.05). **c** Stomatal aperture width was measured in epidermal peels of 4-week-old plants that were treated with MES buffer (control), ABA (10 µM), coronatine (0.5 ng/μl), or ABA (10 µM) and coronatine (0.5 ng/μl) combination for 3 h. The box plots are prepared as described above. Median values are plotted in the boxes with data generated from stomata derived from three independent biological replicates. Statistical analysis was performed by two-way ANOVA followed by Tukey’s test; letters above the bars signify statistically significant differences among groups (*p* ≤ 0.05). Real-time RT-PCR analyses were performed on leaf samples that were dip inoculated with *Pst* DC3000 or *Pst* DC3118 (OD_600 nm_ = 0.2) to determine transcript induction of MYC2 (**d**), ANAC019 (**e**), and ANAC055 (**f**), respectively. Time in hours (h) is indicated. The box plots are prepared as described above. Median values are plotted in the boxes with data generated from three independent biological replicates. Two-way ANOVA with Tukey’s test was performed and asterisks above the bars signify statistically significant differences between *Pst* DC3118 and *Pst* DC3000 treatments (*p* ≤ 0.05). Transcript induction of ANAC019 (**g**) and ANAC055 (**h**) was determined in 2-week-old plants upon treatments with coronatine (0.5 ng/μl) or control using real-time RT-PCR. The box plots are prepared as described above. Median values are plotted in the boxes with data generated from three independent biological replicates. Two-way ANOVA with Bonferroni’s test was performed and asterisks above the bars signify statistically significant differences between L*er* and *atgcn2* upon COR treatments (*p ≤ 0.05)

As a deliberate countermeasure against the host stomatal closure, selected phytopathogenic bacteria, and most prominently a number of *P*. *syringae* strains are able to secret COR to reopen the stomata^35,51^. To examine if AtGCN2 is involved in COR-mediated stomatal reopening, we investigated stomatal immunity using two different types of pathogen infection assays, i.e., syringe pressure infiltration and spray inoculation, and employed *Pst* DC3000 and the COR-deficient mutant strain *Pst* DC3118. In the syringe pressure infiltration assay, bacteria were forcefully delivered at the abaxial side of a leaf through the stomata into intercellular spaces, whereas the bacterial entry through the stomata in the spray inoculation assay was controlled by the natural stomatal immunity^52^. This pair-wise comparison of immune responses allowed us to probe the role of AtGCN2 in COR-dependent stomatal reopening. In the syringe infiltration assay, we detected enhanced disease resistance against *Pst* DC3000 and *Pst* DC3118 in the *atgcn2* mutant (Fig. 3a and Supplementary Fig. 2a, c). In spray inoculation, however, we observed a differential strain-specific response. While the *atgcn2* mutant displayed enhanced resistance against spray inoculation with *Pst* DC3000, this phenotype was lost when *Pst* DC3118 was used (Fig. 3b). To further quantify the responses of *atgcn2* to *P*. *syringae* infection, we conducted a comprehensive analysis of pathogen biomass accumulation in L*er* and *atgcn2* plants following infection with *Pst* DC3000, *Pst* DC3118, and *Pst hrcC* delivered by pressure infiltration or spray on a time course of 4, 12, 24, 36, 48, 60, and 72hpi (Supplementary Fig. 2). This fine-resolution dataset recapitulated our earlier observations done with traditional colony counts at 72 hpi (Fig. 3a, b), and allowed us to glean additional insights into the dynamics of infection as a function of *P*. *syringae* strain and the delivery method. In the pressure-infiltrated plants, we observed differential accumulation of *P*. *syringae* bacteria throughout the course of infection, resulting in markedly lower bacterial loads in the *atgcn2* mutants at 72 hpi (Supplementary Fig. 2a, c, e). In spray-inoculated *atgcn2*, we confirmed the trend of enhanced resistance to *Pst* DC3000 (Fig. 3b and Supplementary Fig. 2b), while the bacterial biomass was not consistently different between L*er* and *atgcn2* sprayed with *Pst* DC3118 (Supplementary Fig. 2d), in concordance with the colony count data (Fig. 3b).

To further solidify our findings, we next tested vacuum infiltration inoculation (equivalent to syringe infiltration, Supplementary Fig. 3a) and dip inoculation (equivalent to spray inoculation, Supplementary Fig. 3b), and we observed consistent trends of bacterial growth in the *atgcn2* mutant. Akin to our *Pst hrcC* results, we detected deficiency of the *atgcn2* mutant in *Pst* DC3118-mediated stomatal closure and *SLAC1* transcript accumulation (Supplementary Fig. 3c, d). Therefore, a direct comparison of stomatal aperture upon *Pst* DC3000 or *Pst* DC3118 challenge was not optimal. Since the initial wave of ABA-induced stomatal closure is intact in the *atgcn2* plants, we decided to adopt an alternate approach, and chose to directly observe the effect of pure COR as well as a combination of ABA and COR on the induction of stomatal reopening. While the control ABA pretreatment effectively closed the stomata in both L*er* and *atgcn2*, the opposite was the case for COR. Unlike in L*er*, the stomata remained closed in the *atgcn2* mutant even in the presence of COR (Fig. 3c) indicating that AtGCN2 is involved in COR-mediated stomatal reopening and consequently the entry of bacteria into the leaf.

Previously, COR was shown to induce the MYC2 TF that in turn upregulates several NAC TFs to confer virulence^51^. To gain additional insights into the mechanism of COR insensitivity in the *atgcn2* mutant, we quantified the *MYC2* transcript after infection with *Pst* DC3000 or *Pst* DC3118. Although not statistically significant, *MYC2* was induced in L*er* plants when challenged with *Pst* DC3000 as compared with *Pst* DC3118, confirming that COR recognition promotes *MYC2* transcription in wild-type plants^48^. However, no induction of *MYC2* was detected in the *atgcn2* mutant when challenged with *Pst* DC3000 (Fig. 3d). Consistent with the previous findings that COR induces NAC TFs through MYC2, we detected a stronger induction of *ANAC019* and *ANAC055* transcripts in the L*er* plants when challenged with *Pst* DC3000 compared with *Pst* DC3118. Akin to *MYC2*, no such effect was observed in the *atgcn2* mutant (Fig. 3e, f). We also analyzed the transcript accumulation of *ANAC019* and *ANAC055* after COR infiltration and found that the *atgcn2* mutant displays a less pronounced transcriptional induction of *ANAC019* and *ANAC055* upon COR treatment compared with the wild-type plants (Fig. 3g, h). Overall, those results collectively demonstrate that AtGCN2 is implicated in COR recognition through the proper regulation of MYC2 and NAC TFs (also see Supplementary Discussion).

### AtGCN2 regulates ABA accumulation and ABA signaling

**Fig. 4:**
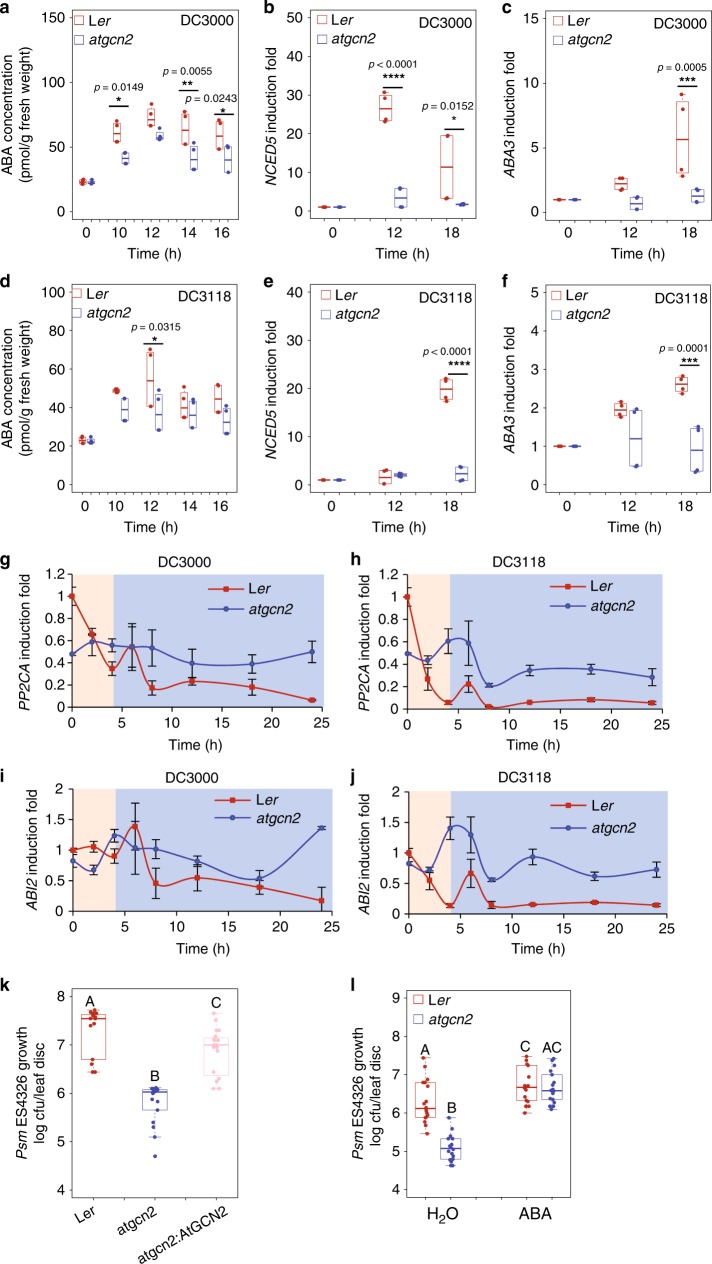
AtGCN2 affects disease susceptibility by promoting ABA accumulation and negatively affecting ABA signaling components accumulation. **a** ABA concentration was determined in 4-week-old plants after *Pst* DC3000 (OD_600nm_ = 0.2) spray inoculation. Median values represent three biological replicates. Two-way ANOVA with Bonferroni’s test was performed; asterisks indicate significant differences compared with wild-type (*p ≤ 0.05 and **p ≤ 0.01). qRT-PCR was performed on 2-week-old plants spray inoculated with *Pst* DC3000 (OD_600nm_ = 0.2) and transcript levels of NCED5 (**b**) and ABA3 (**c**) were quantified. Median values represent three biological replicates. Two-way ANOVA with Bonferroni’s test was performed; asterisks indicate significant differences compared with wild-type (****p *≤* 0.0001 and ***p *≤* 0.001). **d** ABA concentration was determined in 4-week-old L*er* and *atgcn2* plants after *Pst* DC3118 (OD_600nm_ = 0.2) spray inoculation. Median values represent three biological replicates. Two-way ANOVA with Bonferroni’s test was performed; asterisks indicate significant differences compared with wild-type L*er* (*p ≤ 0.05). Real-time RT-PCR analyses were performed on 4-week-old L*er* and *atgcn2* plants spray inoculated with *Pst* DC3118 (OD_600nm_ = 0.2) and transcript levels of NCED5 (**e**) and ABA3 (**f**) were quantified. Median values represent three biological replicates. Two-way ANOVA with Bonferroni’s test was performed; asterisks indicate significant differences compared with wild-type (****p *≤* 0.0001 and ***p *≤* 0.001). Real-time RT-PCR analyses were performed on leaf samples spray inoculated with *Pst* DC3000 (OD_600nm_ = 0.2) (**g**, **i**) or *Pst* DC3118 (OD_600nm_ = 0.2) (**h**, **j**) and transcript levels of PP2CA (**g**, **h**), and ABI2 (**i**, **j**) were quantified. L*er* and *atgcn2* mutants are shown in red and blue lines, respectively. Pink background represents the preinvasive stage while light blue background corresponds to the postinvasive stage. Data represent the mean and SE of three independent biological replicates. **k** Bacterial growth was quantified in 4-week-old *Psm* ES4326 syringe inoculated plants at 3dpi (OD_600nm_ = 0.0001). **l** Bacterial growth was quantified in 4-week-old mock- or 10 mM ABA-treated L*er* and *atgcn2* two days post syringe inoculation (OD_600nm_ = 0.001). Median values represent three biological replicates. Statistical analysis was performed by one-way ANOVA (**k**, **l**) followed by Tukey’s test; letters above the bars signify statistically significant differences among groups (*p* ≤ 0.05)

To investigate additional roles of AtGCN2 in ABA signaling, we first measured changes in the accumulation of total ABA in response to *Pst* DC3000 and *Pst* DC3118. It is known that COR-mediated stomatal re-opening begins at 3 hpi with *Pst* DC3000^50^; thus, we elected to consider the initial 4 h as the preinvasive stage of plant immunity. We did not observe a significant difference in the ABA concentration in wild-type and *atgcn2* in the early phase of pathogen infection with either *Pst* DC3000 and *Pst* DC3118 (Supplementary Fig. 4a, b). However, elevated ABA levels were detected at multiple time points between 10 and 16 h after *Pst* DC3000 challenge in L*er* plants (Fig. 4a), consistent with the upregulation of ABA biosynthesis genes *NCED5* and *ABA3* (Fig. 4b, c). This *Pst* DC3000-induced ABA accumulation was less pronounced in the *atgcn2* plants. Moreover, we also revealed that over-accumulation of ABA between 10 h and 16 h is diminished in the wild-type plants infected with *Pst* DC3118 (Fig. 4d), which correlates with the expression levels of *NCED5* and *ABA3* (Fig. 4e, f). Collectively, these data indicate that COR contributes to over-production of ABA during the late phase of infection with virulent bacterial strains, and AtGCN2 is involved in the regulation of this process. Next, we measured the transcript accumulation of *PP2CA* and *ABI2* in L*er* and *atgcn2* plants upon *Pst* DC3000 and *Pst* DC3118 challenge over the course of 24 hpi and observed that pathogen-triggered transcriptional repression of *PP2CA* and *ABI2* was absent in *atgcn2* (Fig. 4g–j), suggesting that AtGCN2 might indirectly impair the transcription of ABA-negative regulators to benefit pathogen virulence. To test the possible contribution of AtGCN2 to basal disease resistance, we infected wild-type L*er*, *atgcn2*, and a transgenic complementation line expressing functional AtGCN2 under its native promoter (*atgcn2:AtGCN2*) with *Psm* ES4326. We revealed that *atgcn2* displayed enhanced basal disease resistance and exhibited ten times less pathogen growth than L*er*, while the bacterial load in the *atgcn2:AtGCN2* complementation line showed elevated pathogen growth compared with the *atgcn2* mutant (Fig. 4k). On contrary, exogenous application of ABA suppressed the enhanced resistance phenotypes the *atgcn2* mutant, which resulted in the establishment of disease susceptibility levels equal to those of wild-type plants (Fig. 4l). In summary, these data indicate that the virulent bacterial pathogen utilizes AtGCN2 to promote ABA over-accumulation and transcriptional repression of ABA negative regulators, resulting in heightened virulence at the postinvasive stage of infection.

## Discussion

In the present study, we found that bacterial pathogens induce the phosphorylation of eIF2α and this process is dependent on the functional AtGCN2 (Fig. 1a, b and Supplementary Fig. 1c–e). Our previous work identified TBF1, a master immune TF that undergoes a translational derepression following biotic stress. We also previously demonstrated that pathogen infection leads to increased accumulation of both uncharged and charged tRNA^Phe^, as well as induced eIF2α phosphorylation^3^. Given that activation of yeast and mammalian GCN2 is known to result in translational derepression of a key TF^8,9^, and in light of the genetic data presented in this study that provided preliminary insights into a regulatory relationship of AtGCN2 on TBF1 translational derepression (Fig. 1d), it is therefore likely that a similar activation mechanism exists in Arabidopsis. Hence, our study points to a possibility that plants might possess a translational derepression pathway involving AtGCN2, eIF2α, and TBF1 that is triggered during pathogen infection and reminiscent of the yeast GCN2-eIF2α-GCN4 and mammalian GCN2/PERK-eIF2α-ATF4 pathways.

In a recent study, Xu et al. found that AtGCN2-mediated eIF2α phosphorylation is not required for elf18-induced TBF1 translation or disease resistance^53^. GCN2 has the ability to sense and respond to the external amino acid homeostasis, and it is plausible that degradation products of flg22 and elf18, often delivered in excessive concentrations during treatments, could cause amino acid imbalance, indirectly leading to AtGCN2 activation. Thus, we deemed treatments with in vitro synthesized MAMPs inadequate and inconclusive for our experiments.

Moreover, the *atgcn2* mutant allele used in the Xu et al. study is different from the one described in our study. Xu et al. used an allele GABI_862B02 with the T-DNA insertion positioned near the 3′ end of the gene (exon 27). We opted to use a different allele that has the insertion in the gene’s first intron (also described by Lageix et al.^15^).

In another recent study, Izquierdo et al. described roles of Arabidopsis GCN1, GCN2, and GCN20 in various stress responses^45^. However, they failed to detect *P*. *syringae* and SA-induced eIF2α phosphorylation, while we demonstrate that pathogen-mediated eIF2α phosphorylation is dependent upon AtGCN2. Our results are in agreement with a previous report^15^, which showed eIF2α phosphorylation by a wide range of biotic and abiotic treatments including SA. Moreover, they also did not observe any immune phenotypes of *atgcn2* mutant plants, which is contrary to enhanced disease resistance phenotypes to both bacterial (this study) and biotrophic fungal pathogens^24^. We hypothesize that the inability of Izquierdo et al.^45^ to detect eIF2α phosphorylation and immune-related phenotypes of *atgcn2* is possibly due to different immuno-detection methods, type and developmental stage of plant tissues used, and differences between bacterial strains and application methods.

Our results support a function for AtGCN2 in the plant immune system. Recently, it was also shown that global translational reprogramming is a fundamental layer of immune regulation in Arabidopsis^53^ and that the uORFs within the TBF1 mRNA can function autonomously in translational regulation when transformed into rice^54^. What’s more, the GCN2 kinase also contributes to mammalian innate immune responses upon pathogen attack^13,55–57^. The functions of animal GCN2 also span additional aspects of cellular activities, such as regulating inflammation, establishing adaptive immunity and manipulating disease progression. In addition, the nutrient sensing mechanism of the mammalian GCN2 is also linked with mammalian target of rapamycin (mTOR1), which is a central research focus in immunology and biology of aging^58,59^. Moreover, the downstream ATF4 TF in human is directly recruited by TLR4 receptor and required for the inflammatory cytokine production^60^, akin to the plant TBF1 that is a key factor in growth-to-defense transition^3^. This highlights the crucial roles of the GCN2-mediated activation of various defenses and lends additional support in the overall functional conservation of GCN2-controlled immune processes in mammals and plants.

Phytohormones perform essential roles in every aspect of plant life^61^ and engage in a multitude of synergistic and antagonistic interactions at various regulatory levels to maintain cellular homeostasis. Our bioinformatics analysis identified a set of TBF1-dependent differentially expressed genes that are implicated in ABA biosynthesis and signaling. Additional experiments confirmed that two very well defined negative regulators of ABA signaling, PP2CA and ABI2^62^, are misregulated in both *atgcn2* and *tbf1* mutants in response to virulent bacterial pathogens. Likewise, genetic analyses of positive and negative regulators of ABA in conjunction with different methods of pathogen delivery, showed that ABA signaling interacts antagonistically or synergistically with SA or JA to contribute to the plant immune system throughout different infection phases^63^. These previously proposed, opposing functions of ABA are in concordance with our genetic and pathology data. During the late phase of infection with *Pst* DC3000, low accumulation of ABA in *atgcn2* is correlated with a decrease in transcript accumulation of ABA biosynthetic genes *NCED5* and *ABA3* (Fig. 4b, c) and increased *PP2CA* and *ABI2* gene expression (Fig. 4g, i). This suggests that virulent bacterial pathogens might utilize AtGCN2 to potentiate disease susceptibility. It was recently reported that members of the AvrE effector family from *Pst* DC3000 target PP2A complexes in susceptible hosts via direct interaction/association with specific B’ regulatory subunits^64^. Interestingly, Ton et al. previously hypothesized that ABA plays opposing roles in pre and postinvasive phases of pathogen infection^37^. While ABA positively contributes to stomatal immunity (detailed below), virulent pathogens hijack ABA biosynthesis and signaling that in turn suppresses SA-mediated defenses at the later infection stage. It is plausible, therefore, that the lower levels of ABA in the *atgcn2* mutant allow for higher SA accumulation. Consistent with these data, we previously observed a marked increase in the SA biosynthetic gene *SID2* levels in the *atgcn2* plants at 12 and 24 h after SA spray as well as increased levels of resistance to biotrophic powdery mildew fungus *Golovinomyces cichoracearum*^24^. Taken together, we propose that AtGCN2 is a positive regulator of ABA biosynthesis and signaling, and virulent pathogens potentially target AtGCN2 to establish disease susceptibility. Consistent with the previous reports, our data confirm the opposing functions of ABA in different phases of pathogen infection.

In addition to the roles of ABA in postinvasive immunity, a plethora of genetic, biochemical and infection studies in the last decade has demonstrated that the core components of ABA pathway including RCAR, SLAC1, and PP2CA play positive roles in preinvasive immunity^62,65–68^. We provided evidence that AtGCN2 positively contributes to immunity through the ABA signaling, in particular the activities of ion channels in the guard cells (Fig. 2h, i). Stomata, which serve as the gas exchange pores, can alter their aperture in response to environmental cues or pathogen attack^50,69,70^. Recently, SCORD5 (susceptible to coronatine-deficient Pst DC3000), which encodes an ATP-binding cassette protein AtGCN20/AtABCF3, was shown to be involved in regulating stomatal aperture^47^. Intriguingly, the GCN1-GCN20 complex interacts with GCN2 and contributes to activation of downstream translational reinitiation in yeast^42–44^. It remains to be tested whether AtGCN2 and AtGCN20 form a functional complex; however, it was shown that C-terminal region of AtGCN1 (ILITYHIA) interacts with AtGCN2 and contributes to AtGCN2-dependent eIF2α phosphorylation^71^. AtGCN1 and AtGCN4 are also implicated in the regulation of stomatal aperture upon pathogen attack^46–48^. Additional players, such as yeast Yih1 protein and its mammalian ortholog IMPACT, compete with GCN2 for GCN1 binding and inhibit eIF2α phosphorylation in yeast and mammals, respectively^72,73^. However, no such orthologs have been isolated in Arabidopsis. Future genetic and biochemical experiments could identify such novel regulators of AtGCN2 activation and stomatal immunity.

**Fig. 5:**
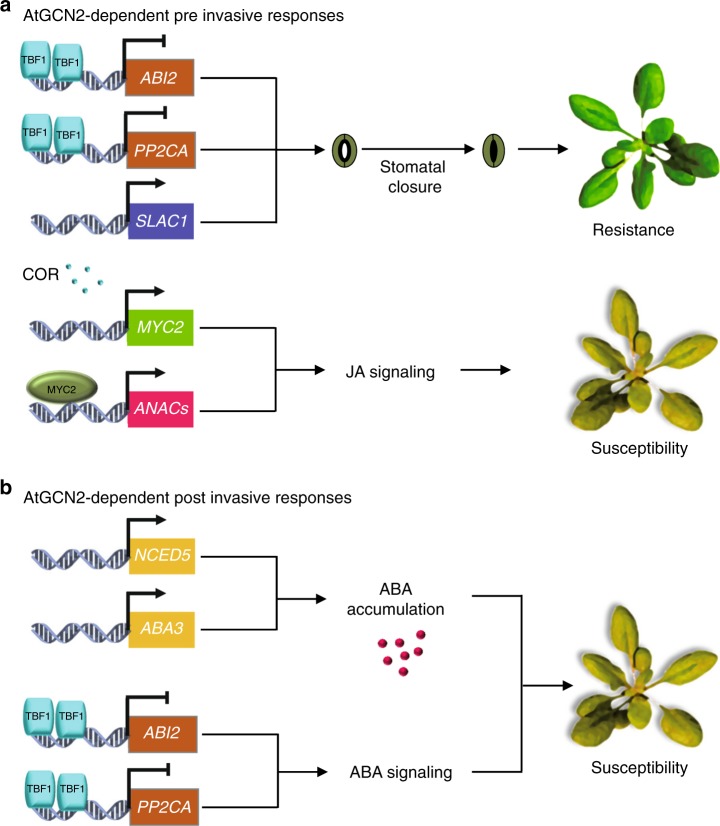
A model representing the key targets of AtGCN2 in immune responses during preinvasive and postinvasive stages of bacterial pathogen infection. **a** AtGCN2 exhibits dual roles in the plant immunity during the preinvasive stage of an infection event. AtGCN2 contributes to plant immunity by enhancing pathogen-triggered stomatal closure through TBF1-mediated repression of negative regulators of ABA signaling, *ABI*2 and *PP2CA*, as well as via upregulation of ion transporter *SLAC1*. Moreover, AtGCN2 is also required for coronatine-mediated virulence by enhancing the expression of the key JA signaling modulator, *MYC2*, and consequently its transcriptional targets *ANAC019* and *ANAC055*. **b** During the postinvasive stage of bacterial infection, AtGCN2 promotes ABA accumulation through indirect positive regulation of ABA biosynthetic genes *NCED5* and *ABA3*, as well as through TBF1-mediated repression of negative regulators of ABA signaling, *ABI2* and *PP2CA*

It is also remarkable that *atgcn2*, despite being affected in ABA signaling during both early and late stages of infection (Fig. 5a, b), can perceive the initial ABA signal and respond correctly with the stomatal closure. Although the ABA receptor-related transcripts are misregulated in the *tbf1* mutant following immune stress (Fig. 2), their basal levels are normal when comparing *tbf1* to Col-0^3^ and we hypothesize that resulting translation of ABA perception and signaling components is sufficient for a successful initial trigger. While a number of ABA signaling genes are misregulated in the *atgcn2* plants, their expression is not completely absent, and early ABA accumulation is unchanged (Supplementary Fig. 4), thus it is likely that un-challenged *atgcn2* plants possess the necessary elements of the ABA perception and signaling machinery. External application of ABA at a concentration much higher than the physiologically active levels may be over-saturating the ABA receptor and signaling pathway to permit successful perception and transduction of the ABA signal and uninterrupted initial wave of ABA-induced stomatal closure (Fig. 3c).

We also demonstrated that AtGCN2 is involved in COR-mediated stomatal reopening, a hallmark of virulent bacterial infection. Upon delivery, COR, a structural mimic of the active form of JA, directly binds to the JA receptor and consequently leads to the derepression of MYC2, a central transcriptional regulator of JA signaling. Among the direct downstream MYC2 targets, a set of NAC TFs was shown to exert inhibitory effects on SA biosynthesis and metabolism^51^. It was recently suggested that MYC2 might be under translational control during seed germination^74^. We demonstrated that AtGCN2 is required for COR recognition through proper regulation of MYC2 and NAC TFs. While COR suppresses the SA-mediated immunity to reopen the stomata, the complete mechanism governing this phenomenon is still lacking. We propose that AtGCN2 is an upstream regulator of this process that may serve as an additional player in hormonal interplay during preinvasive immunity (Fig. 5a, Supplementary Discussion). Collectively, this body of evidence highlights the expanding repertoire of AtGCN2-associated regulators and further supports the recently emerging concept of translational regulation in stomatal immunity.

In conclusion, we discovered AtGCN2 as an immune regulator that triggers eIF2α-mediated downstream signaling events. We gathered evidence indicating a possibility of AtGCN2 being directly or indirectly implicated in translational control of TBF1, which leads to the repression of ABA signaling components upon pathogen infection. We demonstrated the opposing roles of AtGCN2 in regulating ABA accumulation and signaling that contribute to pre and postinvasive plant immunity. Finally, our conclusions suggest a conserved role of GCN2 in various forms of immune responses across kingdoms, highlighting AtGCN2 as a molecule of interest for plant and mammalian immunology.

## Methods

### Plant materials and growth conditions

Wild-type *Arabidopsis thaliana* (L.) Heynh. accessions Columbia-0 and Landsberg *erecta* (L*er*) were used in this study. The *atgcn2* Genetrap insertion line GT8359 (L*er* background) was obtained from Cold Spring Harbor Laboratory, New York. The *tbf1* T-DNA insertion line SALK_104713 was obtained from ABRC. *Agrobacterium tumefaciens* carrying *uORF1-uORF2-GUS* or *uorf1-uorf2-GUS* was used to transform L*er* and the *atcgn2* plants to generate stable Arabidopsis transgenic lines as described previously^3^. Plants were grown on Super Fine Germination Mix soil (Sun Gro Horticulture) under a 12 h light/12 h dark photoperiod (21 °C, 100 μmol/m^2^/s light intensity and 40% relative humidity).

### Pathogen strains

*Pseudomonas syringae* pv. *maculicola* ES4326 (*Psm* ES4326), *Psm* ES4326 carrying avirulent effector avrRpm1, *P. syringae* pv. *tomato* DC3000 (*Pst* DC3000), *Pst* DC3118 defective in COR production and *Pst* DC3000 *hrcC* defective in TTSS-mediated effectors delivery were used in this study.

### Pathogen infection and quantification assays

For quantifying disease resistance during postinvasive stage, 4-week-old plants were syringe infiltrated with *Pst* DC3000 (OD_600nm_ = 0.0002) or *Pst* DC3118 (OD_600nm_ = 0.0002) and bacterial growth was quantified three days post inoculation as described previously^75^. For characterizing the defense response during the postinvasive stage in early developmental stage, 2-week-old plants were vacuum inoculated with *Pst* DC3000 (OD_600nm_ = 0.002) or *Pst* DC3118 (OD_600nm_ = 0.002) with 0.002% Silwet L-77 and bacterial growth was quantified 2 days post inoculation. For characterizing the defense response during the preinvasive stage, 4-week-old plants were spray inoculated with *Pst* DC3000 (OD_600nm_ = 0.2) or *Pst* DC3118 (OD_600nm_ = 0.2) with 0.02% Silwet L-77 and pathogen growth was quantified 3 days post inoculation. For characterizing the defense response during the preinvasive stage in plants at an early developmental stage, 2-week-old plants were dip inoculated with *Pst* DC3000 (OD_600nm_ = 0.05) or *Pst* DC3118 (OD_600nm_ = 0.05) with 0.02% Silwet L-77 and pathogen growth was quantified 2 days post inoculation. For pathogen biomass quantification by qPCR, 4-week-old plants were inoculated with *Pst* DC3000, *Pst* DC3000 *hrcC*, or *Pst* DC3118 with OD_600 nm_ = 0.0002 (syringe infiltration) or OD_600nm_ = 0.2 with 0.02% Silwet L-77 (spray), and DNA was extracted from leaf tissue at specific time points by grinding tissue in 200 μl CTAB Extraction Buffer (2% cetyl trimethylammonium bromide, 100 mM Tris-HCl pH 8, 1.4 M NaCl, 20 mM EDTA, 0.5% β-Mercaptoethanol, 2% polyvinyl pyrrolidone). For qPCR analysis, about 10 ng of template DNA were used and bacterial biomass was measured using *P*. *syringae*-specific oprF primer pair^76^ (Table S2) using GoTaq qPCR master mix (Promega) in a RealPlex S MasterCycler (Eppendorf).

### Gene expression analysis

Total RNA was extracted from Arabidopsis leaves using a TRIzol reagent (Invitrogen), and genomic DNA contamination was removed by DNase I (Ambion) treatment. Reverse transcription was conducted with the SuperScript III first-strand RT-PCR kit (Invitrogen), and gene expression was determined using GoTaq qPCR Master Mix (Promega) with transcript-specific primers in a RealPlex S MasterCycler (Eppendorf). Primers used for qRT-PCR are listed in the Supplementary Table 2.

### β-glucuronidase (GUS) activity quantification

Transgenic T_3_ homozygous lines uORF1-uORF2-GUS (L*er*), uORF1-uORF2-GUS (*atgcn2*), and uorf1-uorf2-GUS (L*er*) were syringe infiltrated with *Psm* ES4326/avrRpm1 (OD_600nm_ = 0.1), and tissues were collected at specified time points. Total proteins were extracted with extraction buffer (50 mM NaPO_4_ [pH 7.0], 1 mM Na_2_EDTA, 0.1% SDS, 0.1% Triton X-100, protease inhibitor for plant extracts [Sigma], and 10 mM β-mercapethanol). As described previously^3^, GUS activity was quantified by incubating protein extract with 1 mM MUG (4-methylumbelliferyl β-D-glucuronide). The reaction was terminated with 1 M Na_2_CO_3_ and fluorescence was measured using a microplate Reader (Tecan) with excitation wavelength of 365 nm, an emissions wavelength of 455 nm and a filter wavelength of 430 nm. The relative GUS activity was obtained by normalizing data to the Bradford assay.

### Protein extraction and immunoblot analysis

Total proteins were extracted from liquid MS-grown 2-week-old seedlings exposed to mock or live pathogen *Pst* DC3000, *Pst* DC3118, or *Pst* DC3000 *hrcC* (OD_600nm_ = 0.02) by grinding tissue in 200 μl extraction buffer (10 mM HEPES [pH 7.7], 2 mM EDTA, 150 mM NaCl, 10 mM MgCl_2_, 0.3% Triton X-100, protease inhibitor for plant extracts (Sigma), proteasome inhibitor MG132 (Sigma) and PhoSTOP (Roche)^3^). Protein extracts were separated on 10% SDS-PAGE gel and proteins were transferred onto nitrocellulose membrane (Whatman) with the semi-dry transfer system (FisherBiotech). Equal protein loading was confirmed by 0.1% Ponceau S staining. To detect the phosphorylation of eIF2α, membranes were blocked in 5% skimmed milk in TBS-T buffer (10 mM Tris–HCl [pH 7.5], 150 mM NaCl and 0.1% Tween 20) for 1 h at room temperature before incubation with 1:1000 dilution of primary anti-eIF2αS1 antibody (Abcam) overnight at 4 °C. For detection of total eIF2α protein, polyclonal antibody against peptide (IRRRMTPQPMKIRAD) was raised in rabbits (Genscript)^18^. Membranes were blocked in 5% skimmed milk in TBS-T buffer overnight at 4 °C and incubated with 1:4000 dilution of primary antibody in 2% skimmed milk in TBS-T buffer. After incubation with primary antibodies, membranes were incubated with 1:5000 dilution of secondary anti-rabbit HRP-conjugated antibody (Santa Cruz Biotechnology) in 2% skimmed milk in TBS-T buffer for 1 h at room temperature. Immunoblots were detected with Clarity ECL Substrate (Bio-Rad) for chemiluminescence development.

### ABA quantification

Four-week-old plants were spray inoculated with *Pst* DC3000 or *Pst* DC3118 (OD_600nm_ = 0.2) with 0.02% Silwet L-77 and ABA extraction was performed as described previously^77^. ABA was extracted using extraction buffer (10 mM HCl, 1% PVPP in methanol) overnight at 4 °C. The tissue extract was neutralized with 1 M NaOH before dried under the SpeedVac (Thermofisher). The dried residues were resuspended in water and ABA concentration was measured using the Phytodetek Immunoassay kit for ABA (AGDIA Inc.).

### The stomatal aperture assay

To measure the stomatal aperture, epidermal leaf peels of the abaxial side were collected from mature leaves of 4-week-old plants. Leaf peels were incubated in MES buffer (25 mM MES [pH 6.15] and 10 mM KCl) or MES buffer containing 10 µM ABA (Sigma) or 0.5 ng/μl COR (Sigma) on the top of a glass slide and observed under the light microscope at specified time points^50^. Stomatal response to living bacteria was performed by incubating leaf peels in water or water containing *Pst hrcC* (OD_600 nm_ = 0.2) (OD_600 nm_ = 0.2) and observed at specified time points. Random (in order to avoid personal preference) pictures were taken under the microscope and at least 60 stomata were recorded for each treatment per time point. The stomatal aperture measurement was performed with NIS element software (https://www.nikoninstruments.com/Products/Software/NIS-Elements-Advanced-Research/NIS-Elements-Viewer).

### Statistics and reproducibility

Statistical differences were calculated by two-way ANOVA or one-way ANOVA followed by Tukey’s test or Bonferroni’s test in a GraphPad Prism 8. Statistically significant differences are indicated with *p < 0.05, **p < 0.01, ***p < 0.001, and ****p < 0.0001. Raw data used to create graphs are available in Supplementary Data.

### Reporting summary

Further information on research design is available in the Nature Research Reporting Summary linked to this article.

## Supplementary information

Supplementary Information

Description of Additional Supplementary Files

Supplementary Data

Reporting Summary

## Data Availability

The *tbf1* microarray data have been published previously and are available in the Gene Expression Omnibus (GEO) database under the accession number GSE34047. All other relevant data are available in the paper, Supplementary Information files, and from the corresponding author upon request.
