# Supplementary material for: Arabidopsis GCN2 kinase contributes to ABA homeostasis and stomatal immunity
Source: Commun Biol. 2019 Aug 8;2:302. doi: 10.1038/s42003-019-0544-x (PMC6687712; doi:10.1038/s42003-019-0544-x)
Supplement: Supplementary file 1 — Supplementary Information [file 42003_2019_544_MOESM1_ESM.pdf]

Supplementary Figures

a

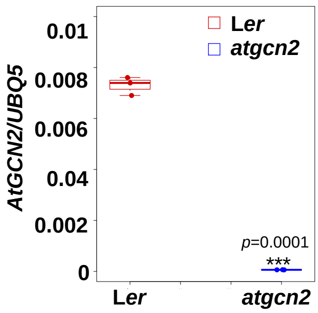

c

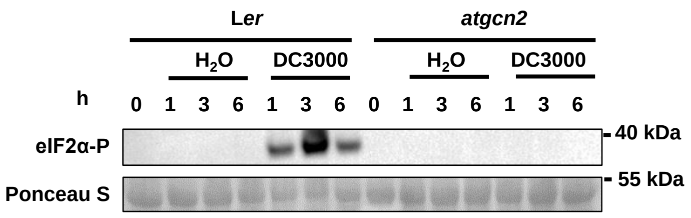

b

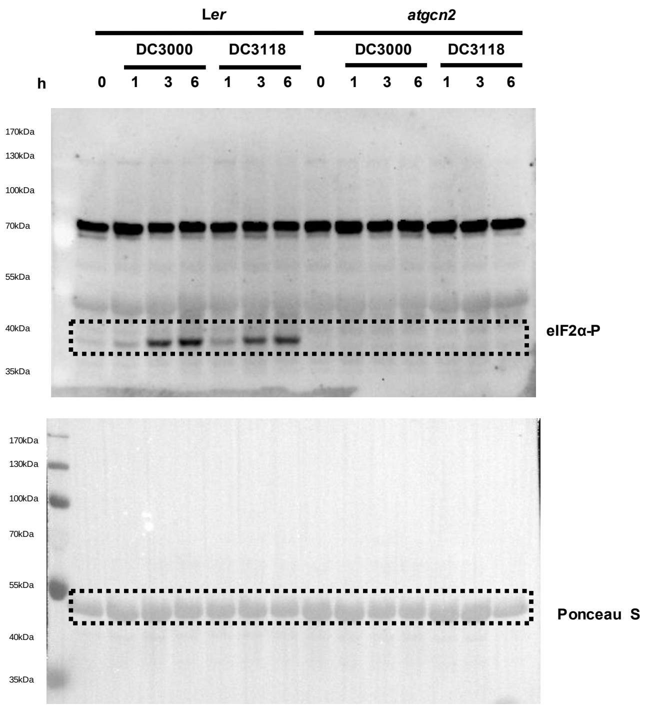

d

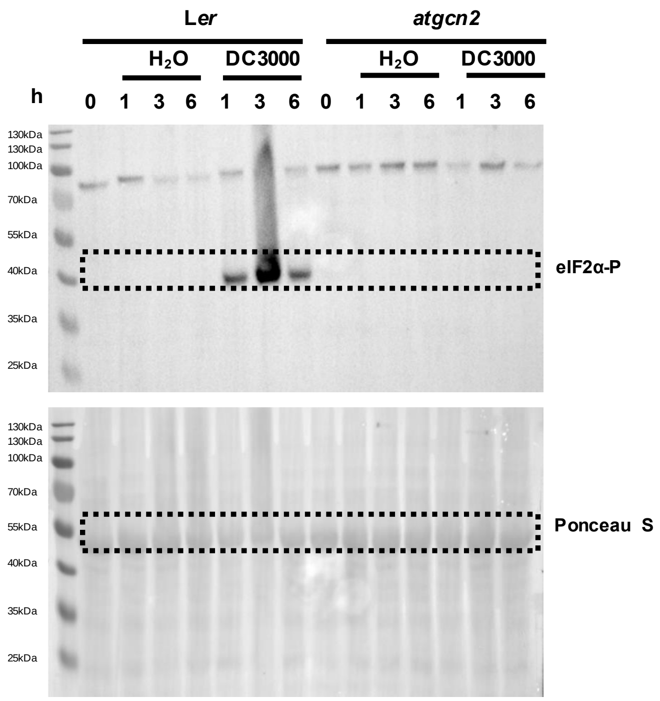

e

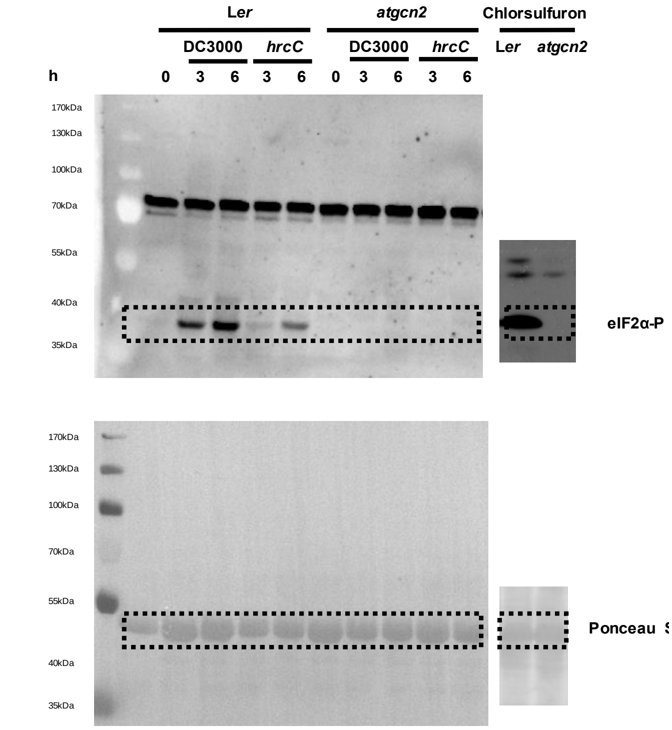
Ler
atgcn2
Chlorsulfuron
DC3000
hrcC
DC3000
hrcC
Ler
atgcn2
h
0 3 6 3 6 0 3 6 3 6
170kDa
130kDa
100kDa
70kDa
55kDa
40kDa
35kDa
eIF2α-P
Ponceau S

f

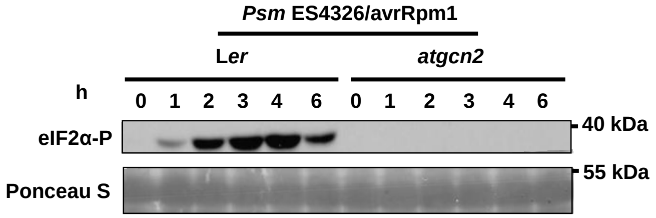
Psm ES4326/avrRpm1
Ler
atgcn2
h
0 1 2 3 4 6 0 1 2 3 4 6
eIF2α-P
Ponceau S
40 kDa
55 kDa

g

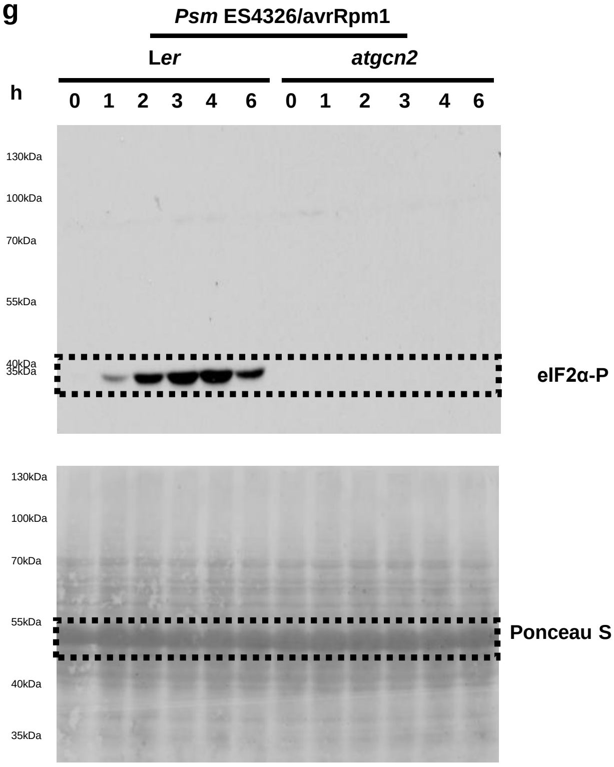
Psm ES4326/avrRpm1
Ler
atgcn2
h
0 1 2 3 4 6 0 1 2 3 4 6
130kDa
100kDa
70kDa
55kDa
40kDa
35kDa
eIF2α-P
Ponceau S

h

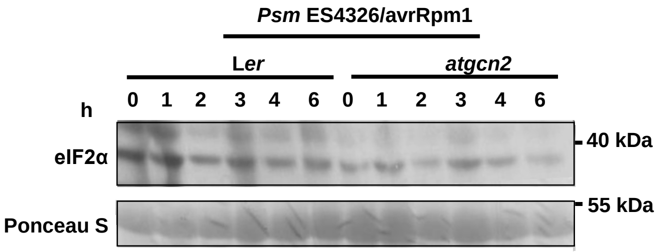
Psm ES4326/avrRpm1
Ler
atgcn2
h
0 1 2 3 4 6 0 1 2 3 4 6
eIF2α
Ponceau S
40 kDa
55 kDa

i

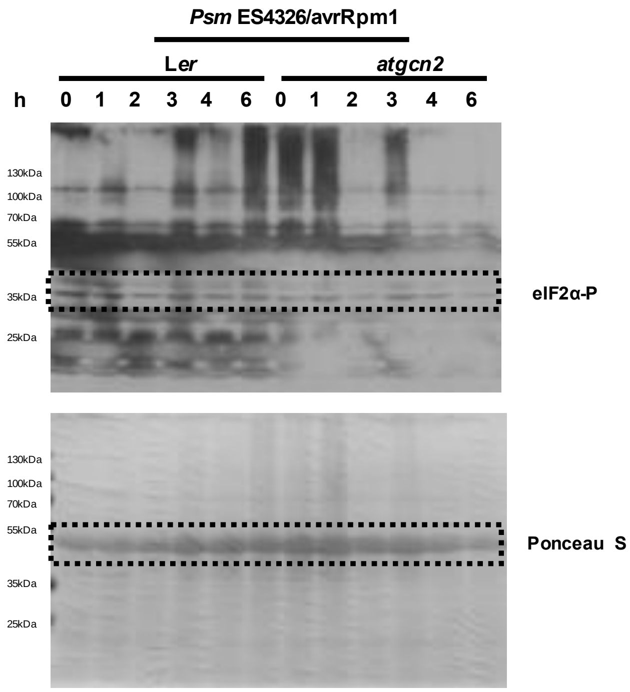
Psm ES4326/avrRpm1
Ler
atgcn2
h
0 1 2 3 4 6 0 1 2 3 4 6
130kDa
100kDa
70kDa
55kDa
35kDa
25kDa
eIF2α-P
Ponceau S

j

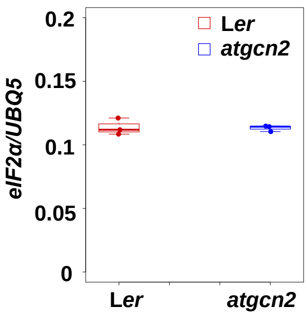
0.2
0.15
0.1
0.05
0
eIF2α/UBQ5
Ler
atgcn2

k

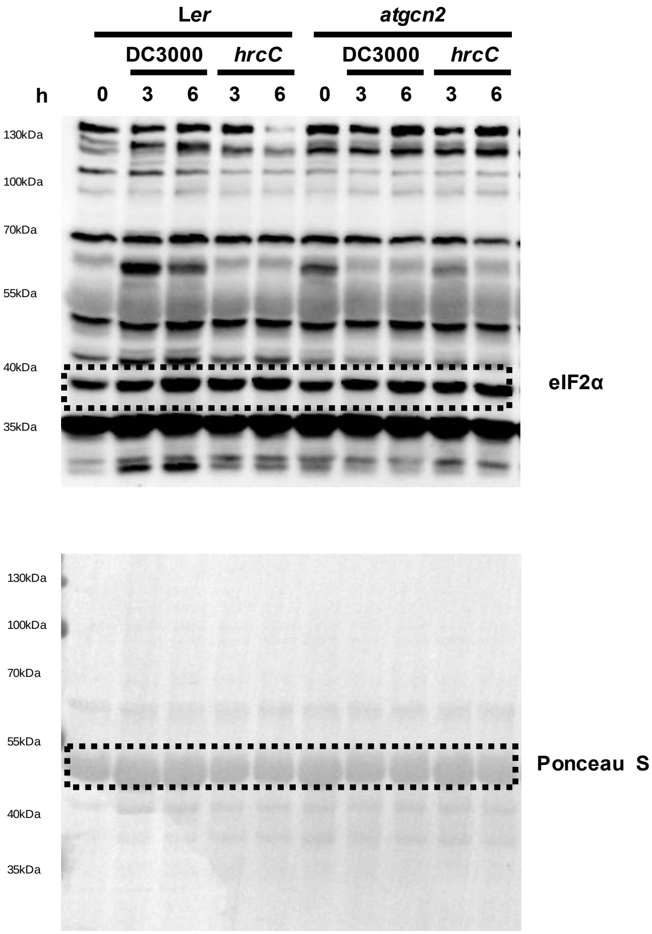

Ler
atgcn2
DC3000
hrcC
DC3000
hrcC
h
0
3
6
3
6
0
3
6
3
6
130kDa
100kDa
70kDa
55kDa
40kDa
35kDa
eIF2α
Ponceau S

l

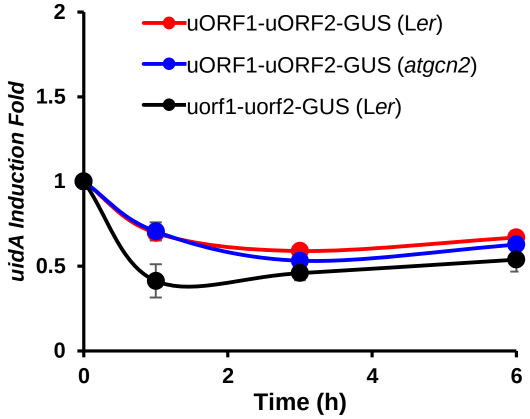

uORF1-uORF2-GUS (Ler)
uORF1-uORF2-GUS (atgcn2)
uorf1-uorf2-GUS (Ler)
uidA Induction Fold
Time (h)

m

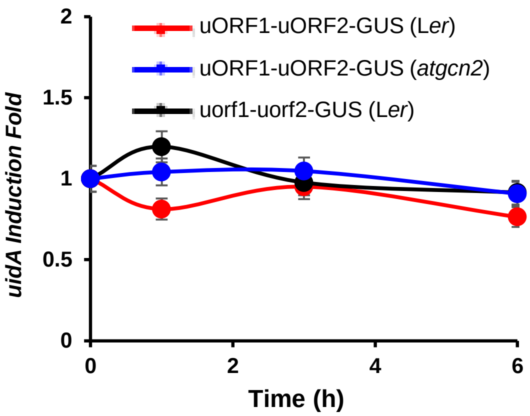

uORF1-uORF2-GUS (Ler)
uORF1-uORF2-GUS (atgcn2)
uorf1-uorf2-GUS (Ler)
uidA Induction Fold
Time (h)

Supplementary Figure 1. AtGCN2 is essential for pathogen-triggered eIF2α phosphorylation without affecting *TBF1* transcript accumulation.

a. Transcript accumulation of *AtGCN2* was measured in four-week-old plants by real-time RT-PCR. The box plots extend from the 25th to 75th percentiles and the whiskers extend from the minimum to the maximum level. Median values are plotted in the box with data generated from three independent biological replicates, each of them representing means of two technical replicates. Statistical analysis was performed with Student's t-test, p-value is listed. **b.** Detection of phosphorylated form of eIF2α in the samples prepared from two-week-old plants treated with *Pst* DC3000 or *Pst* DC3118 (OD_{600nm} = 0.02) at the indicated time points in hours (h) (full blots for Fig. 1a). Phosphorylation state-specific (S51) anti-human eIF2α antibody was used. Ponceau S staining shows loading amounts. **c.** Detection of phosphorylated form of eIF2α in the samples prepared from two-week-old plants treated with *Pst* DC3000 (OD_{600nm} = 0.02) or control H_2O at the indicated time points in hours (h). Phosphorylation state-specific (S51) anti-human eIF2α antibody was used. Ponceau S staining was used to determine loading. **d.** Full blots for Supplementary fig. 1c. **e.** Detection of phosphorylated form of eIF2α in the samples prepared from two-week-old plants treated with *Pst* DC3000 or *Pst* hrcC (OD_{600nm} = 0.02) at the indicated time points in hours (h) (full blots for Fig. 1b). Phosphorylation state-specific (S51) anti-human eIF2α antibody was used. Ponceau S staining shows loading amounts. **f.** Detection of phosphorylated form of eIF2α in the samples prepared from two-week-old plants treated with *Psm* ES4326/avrRpm1 (OD_{600nm} = 0.02) at the indicated time points in hours (h). Phosphorylation state-specific (S51) anti-human eIF2α antibody was used. Ponceau S staining was used to determine loading. **g.** Full blots for Supplementary fig. 1f. **h.** Time course analysis of total eIF2α protein accumulation in two-week-old plants upon *Psm* ES4326/avrRpm1 (OD_{600nm} = 0.02) challenge. Time points are shown in hours (h). Ponceau S staining was used to determine loading. **i.** Full blots for Supplementary fig. 1h. **j.** Transcript accumulation of *eIF2α* was measured in four-week-old plants by real-time RT-PCR. The box plots extends from the 25th to 75th percentiles and the whiskers extend from the minimum to the maximum level. Median values are plotted in the box with data generated from three independent biological replicates, each of them represented as means of two technical replicates. Statistical analysis was performed with Student's t-test and no significant difference was detected. **k.** Time course total eIF2α protein accumulation in two-week-old plants upon *Pst* DC3000 or *Pst* hrcC (OD_{600nm} = 0.02) challenge. Time points are shown in hours (h) (full blots for Fig. 1c). Ponceau S staining shows loading amounts. **l.** Transcript accumulation of *iudA* (encoding the GUS reporter) was measured by real-time RT-PCR in four-week-old transgenic T_3 plants uORF1-uORF2-GUS (L*er*), uorf1-uorf2-GUS (L*er*) and uORF1-uORF2-GUS (*atgcn2*) treated with *Pst* DC3000 (OD_{600nm} = 0.02), sampled at indicated time points. Data represent the mean and standard error of three independent biological replicates. **m.** Transcript accumulation of *iudA* (encoding the GUS reporter) was measured by real-time RT-PCR in four-week-old transgenic T_3 plants uORF1-uORF2-GUS (L*er*), uorf1-uorf2-GUS (L*er*) and uORF1-uORF2-GUS (*atgcn2*) treated with *Psm* ES4326/avrRpm1 (OD_{600nm} = 0.02), sampled at indicated time points. Data represent the mean and standard error of three independent biological replicates.

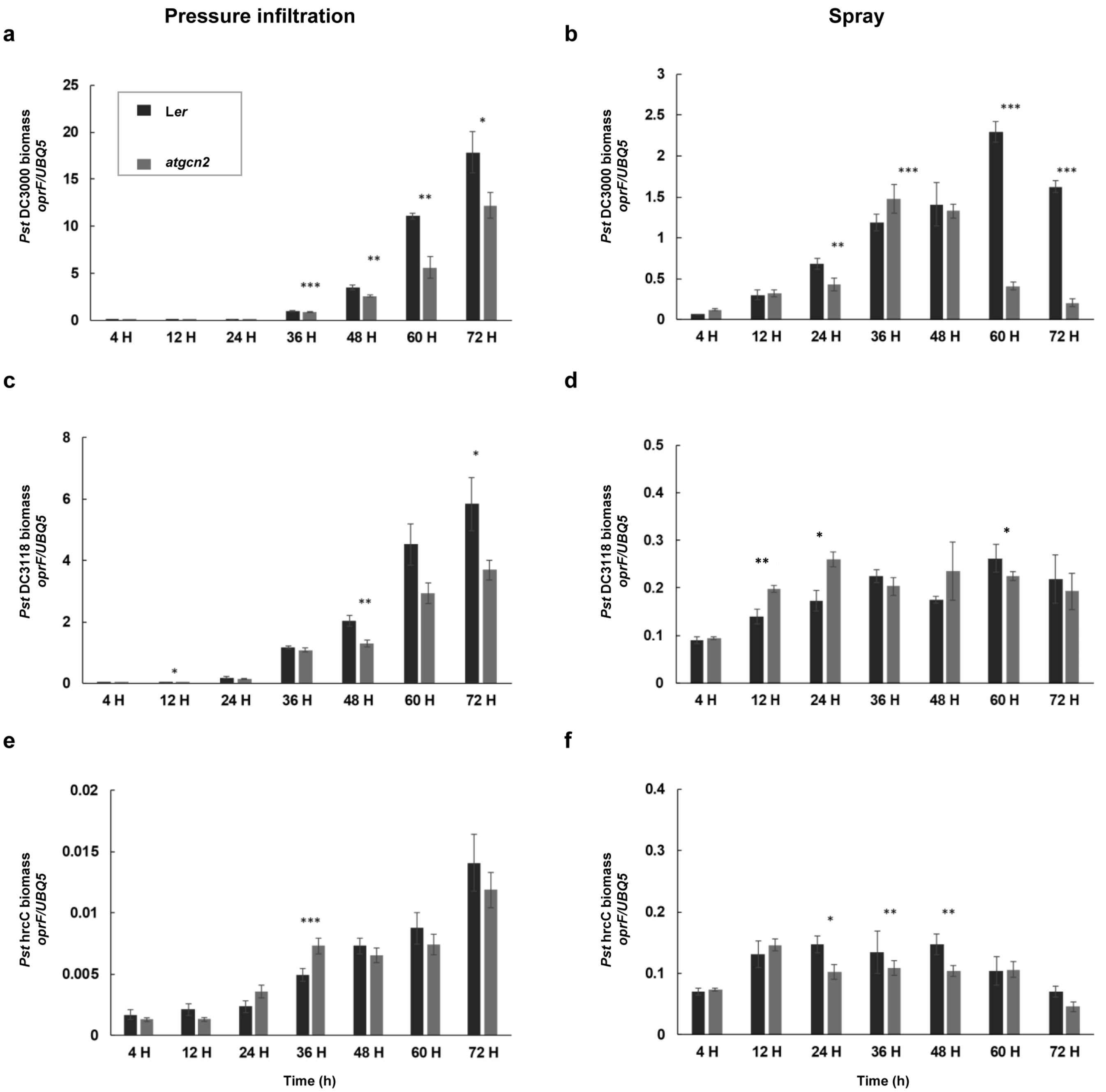

Supplementary Figure 2. Time-course quantification of *P. syringae* biomass. L*er* and *atgcn2* plants were infected with *Pst* DC3000 (**a**, **b**)*, Pst* DC3118 (**c**, **d**), or *Pst* DC3000 *hrcC* (**e**, **f**) by pressure infiltration (OD_{600nm}=0.0002) (**a**, **c**, **e**) or spray (OD_{600nm}=0.2) (**b**, **d**, **f**). Pathogen growth were quantified in four weeks old infected plants at indicated time points (4, 12, 24, 36, 48, 60, and 72hpi) by qPCR analyses. Data represent the mean and standard error of three independent biological replicates. Two sample t-test was performed for statistical analysis, asterisks indicate significant differences compared to wild-type L*er* (* p<0.05, ** p<0.01,***p<0.001).

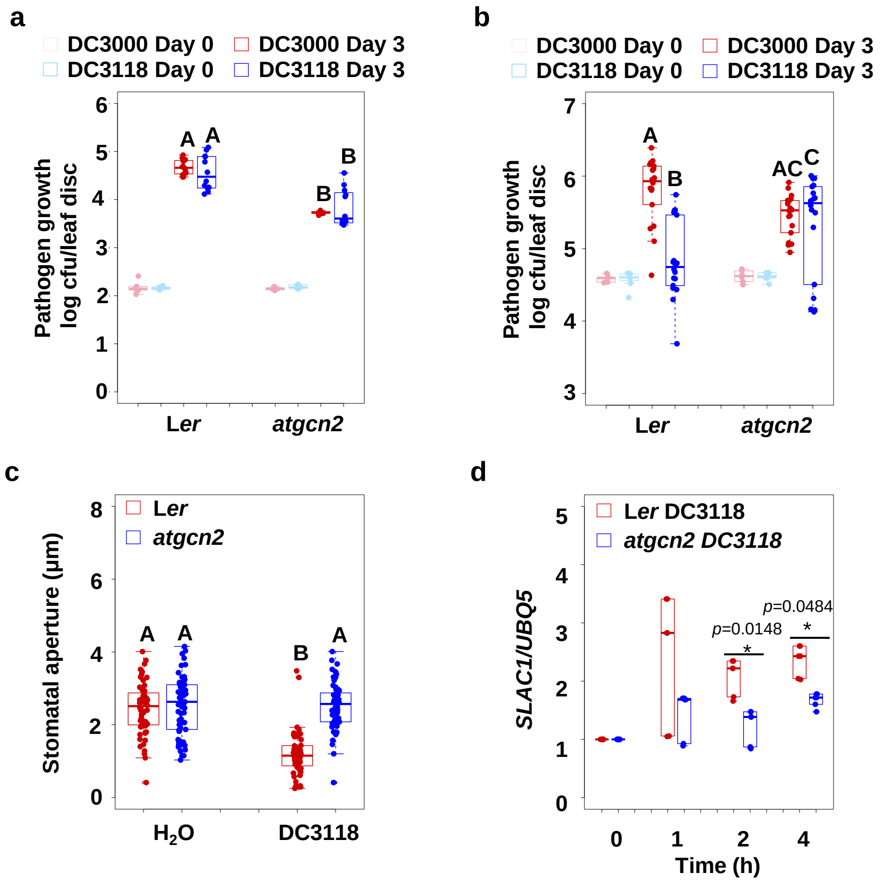

Supplementary Figure 3. *atgcn2* mutant is deficient in pathogen-triggered stomatal responses.

a-b. Pathogen growth was quantified in two-week-old plants infected with *Pst* DC3000 or *Pst* DC3118 using vacuum inoculation (OD_{600nm} = 0.002 and 0.002% Silwet L-77) (**a**) and dip inoculation (OD_{600nm} = 0.05 and 0.02% Silwet L-77) (**b**). The box plots extend from the 25th to 75th percentiles and the whiskers extend from the minimum to the maximum level. Median values are plotted in the box with data generated from three independent biological replicates. Statistical analysis was performed by two-way ANOVA followed by Tukey's test, letters above the bars signify statistically significant differences among groups (p≤0.05). **c.** Stomatal aperture width was measured in epidermal peels of four-week-old plants that were treated with *Pst* DC3118 (OD_{600nm} = 0.2) for three hours. The box plots extend from the 25th to 75th percentiles and the whiskers extend from the minimum to the maximum level. Median values are plotted in the box with data generated from 180 stomata derived from three independent biological replicates. Two-way ANOVA with Tukey's test was performed, letters above the bars signify statistically significant differences among groups (p≤0.05). **d.** Transcript induction of *SLAC1* was determined in four-week-old plants upon treatments with *Pst* DC3118 (OD_{600nm} = 0.2) spray inoculation using real-time RT-PCR. The box plots extend from the 25th to 75th percentiles and the whiskers extend from the minimum to the maximum level. Median values are plotted in the box with data generated from three independent biological replicates. Two-way ANOVA with Tukey's test was performed, asterisks indicate significant differences compared to wild-type L*er* (***p≤0.001).

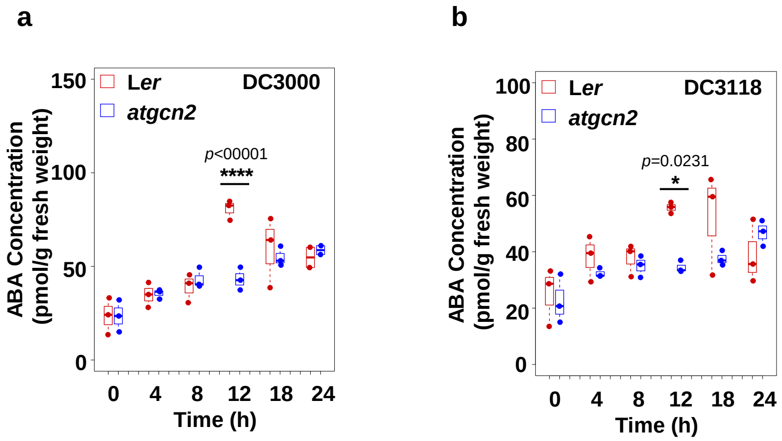

Supplementary Figure 4. AtGCN2 affects ABA accumulation upon pathogen infection

a-b. ABA concentration was determined in two-week-old L*er* and *atgcn2* at the indicated time points after *Pst* DC3000 (OD_{600nm} = 0.2) (**a**) or *Pst* DC3118 (OD_{600nm} = 0.2) (**b**) dip inoculation. The box plots extend from the 25th to 75th percentiles and the whiskers extend from the minimum to the maximum level. Median values are plotted in the box with data generated from three independent biological replicates, each of them shown as means of four technical replications. Two-way ANOVA with Tukey's test was performed, asterisks indicate significant differences compared to wild-type L*er (*p-values are listed).

Supplementary Tables

	AGI	Gene Model Description	# TL1 degenerate	# TL1 exact
Induced by TBF1	AT3G20310	ERF/AP2 transcription factor family (ATERF-7), involved in ABA-mediated responses.	4	0
	AT5G46790	a member of the PYR/PYL/RCAR family proteins and mediate ABA-dependent regulation of protein phosphatase 2Cs ABI1 and ABI2.	2	2
	AT2G40330	a member of the PYR/PYL/RCAR family proteins and mediate ABA-dependent regulation of protein phosphatase 2Cs ABI1 and ABI2.	0	0
	AT5G01560	LecRKA4.3 which negative regulates ABA response in seed germination.	0	0
	AT5G05440	a member of the PYR/PYL/RCAR family proteins and mediate ABA-dependent regulation of protein phosphatase 2Cs ABI1 and ABI2.	0	0
Repressed by TBF1	AT3G57530	Calcium-dependent Protein Kinase and regulates the ABA-responsive gene expression via ABF4.	6	0
	AT5G66880	a member of SNF1-related protein kinases (SnRK2) in the ABA signaling during seed germination, dormancy and seedling growth.	4	0
	AT1G72450	jasmonate-zim-domain protein 6 (JAZ6).	2	2
	AT3G11410	Encodes protein phosphatase 2C. Negative regulator of ABA signalling.	2	2
	AT3G62030	nuclear-encoded chloroplast stromal cyclophilin CYP20-3 (also known as ROC4) which is modulated in response to ABA.	2	0
	AT5G02240	Protein is tyrosine-phosphorylated and its phosphorylation state is modulated in response to ABA in Arabidopsis thaliana seeds.	2	2
	AT5G24030	The SLAH3 protein has similarity to the SLAC1 protein involved in ion homeostasis in guard cells.	2	0
	AT5G63980	Encodes a bifunctional protein that is involved in the response to cold, drought (negative regulator of drought tolerance), and ABA.	2	0
	AT1G01260	basic helix-loop-helix (bHLH) DNA-binding superfamily protein shows similiarity to ABA-inducible transcription factor (AT2G46510.1).	0	0
	AT1G17380	jasmonate-zim-domain protein 5 (JAZ5).	0	0
	AT1G32640	MYC2	0	0
	AT1G70700	jasmonate-zim-domain protein 9 (JAZ9).	0	0
	AT1G75380	nucleases AtBBD1 involved in ABA-mediated callose deposition.	0	0
	AT2G05710	ACO3 is tyrosine-phosphorylated and its phosphorylation state is modulated in response to ABA in Arabidopsis thaliana seeds.	0	0
	AT2G18960	a plasma membrane proton ATPase which affect stomatal closure towards drought and ABA.	0	0
	AT2G33380	Encodes a calcium binding protein whose mRNA is induced upon treatment with NaCl, ABA and in response to dessication.	0	0
	AT2G34600	jasmonate-zim-domain protein 7 (JAZ7).	0	0
	AT2G46510	Encodes a nuclear localized BLH domain containing transcriptional activator involved in response to ABA.	0	0
	AT3G17860	jasmonate-zim-domain protein 3 (JAZ3).	0	0
	AT3G50500	SNRK2.2 which isinvolved in the ABA signaling during seed germination, dormancy and seedling growth.	0	0
	AT3G55610	encodes delta 1-pyrroline-5-carboxylate synthetase B. Gene expression is induced by dehydration, high salt and ABA.	0	0
	AT4G38970	Protein is tyrosine-phosphorylated and its phosphorylation state is modulated in response to ABA in Arabidopsis thaliana seeds.	0	0
	AT5G20900	jasmonate-zim-domain protein 12 (JAZ12)	0	0
	AT5G25610	responsive to dehydration 22 (RD22) mediated by ABA	0	0
	AT5G67030	ABA1 which functions in first step of the biosynthesis of the abiotic stress hormone abscisic acid (ABA).	0	0

Supplementary Table 1. Summary of genes associated with ABA response whose transcription is TBF1-dependent upon elf18 challenge. Numbers of exact and degenerate sequence variants corresponding to TL1 (Translocon 1; TBF1 binding site) are shown to the right.

Primer name	Sequence 5' -> 3'	Application
AtGCN2-F	CAACACTTTCCCGTTTGCAG	qPCR
AtGCN2-R	GTTGACACTGCACCTGAGTAG	qPCR
eIF2α-F	ACTCACAACTCACACCCATTAC	qPCR
eIF2α-R	TTCCCTCATCACCACTCATTTC	qPCR
TBF1-F	GTTGGTTCGCCTTCTG	qPCR
TBF1-R	CCACACCCCAAACAAT	qPCR
PP2CA-F	AAGATCGGTACGACGTCGGTTTGT	qPCR
PP2CA-R	TCTGCACTTCTCCGCAACATGAGA	qPCR
ABI2-F	ACACGTGGCAAGAGAAGTGGAAGA	qPCR
ABI2-R	CCGCAATTCGCGACAAAGATGTGA	qPCR
FRK1-F	AAGATGGCGACTTCG	qPCR
FRK1-R	GCAGGTTGGCCTGTAA	qPCR
SLAC1-F	CCGGGCTCTAGCACTCA	qPCR
SLAC1-R	TCAGTGATGCGACTCTT	qPCR
MYC2-F	CAAGGAGGAGTGTTTGGGATGC	qPCR
MYC2-R	GTCGAAAAATTAAGTTCTCGGGAG	qPCR
ANAC019-F	GCATCTCGTCGCTCAG	qPCR
ANAC019-R	CTCGACTTCCTCCTCCG	qPCR
ANAC055-F	GCGCTGCCTCATAGTC	qPCR
ANAC055-R	CGAGGAATCCCCTCAGT	qPCR
NCED5-F	CCTCCGTTAGTTTCACCAACACT	qPCR
NCED5-R	GGTGTGTCGGAGACGGAGTT	qPCR
ABA3-F	TCCTGAAGATTACAGTTGCTTATTCAC	qPCR
ABA3-R	TGGGTCCACGGAAAAGTCTCT	qPCR
oprF-F	AACTGAAAAACACCTTGGGC	qPCR
oprF-R	CCTGGGTTGTTGAAGTGGTA	qPCR
UBQ5-F	GTAAACGTAGGTGAGTCC	qPCR
UBQ5-R	GACGCTTCATCTCGTCC	qPCR

Supplementary Table 2. List of primers used in this study.

Supplementary Discussion

Jasmonate signaling is an intrinsic part of the SA-ABA crosstalk, thus we examined the expression of key JA signaling markers, such as MYC2, ANAC019 and ANAC055, in the *atgcn2* plants challenged with *Pst* DC3000 and *Pst* DC3118. All of these genes were shown to be highly dependent of COR for their induction. Our results demonstrate that functional AtGCN2 is required for the full induction of MYC2 expression following *Pst* DC3000 infection, indicating that GCN2 action may be required for direct or indirect regulation of one of the components of the COR perception and/or signaling module. Intriguingly, however, the *atgcn2* mutants can markedly induce MYC2 expression when infected with COR-deficient *Pst* DC3118 strain. We hypothesize that an alternative mechanism of MYC2 transcriptional activation might exist in the absence of COR. In fact, a number of recent reports exist that provide evidence that COI1-JAZ action can be uncoupled from coronatine. Ueda et al. 2017 showed that COR can also counter stomatal defense through a COI1-JAZ-independent and ER-mediated function[1]. Cui et al. (2018) demonstrated that EDS1 in complex with its partner PAD4 inhibits MYC2, forming a signaling circuit that blocks actions of coronatine[2]. The initial activation of JA-responsive genes during ETI is dependent on SA and SA receptors NPR3 and NPR4, but not the canonical JA receptor COI1[3]. Geng et al. (2012) showed COR is able to suppress callose deposition in the leaves of *coi1* mutant plants and argued that COR may have multiple targets inside plant cells[4]. While JA is commonly known as a strong inducer of MYC2 expression, Takahashi et al. (2007) showed that JA can also suppress MYC2 transcription via novel MKK3-MPK6 pathway, indicating that there is more than one mechanism of MYC2 regulation and adding another layer of complexity to the JA-COI1-JAZ-MYC2 web[5].

From the host perspective, the ability to suppress the action of coronatine on stomata without impairing COI1-JAZ-dependent plant defense responses would be highly desirable in an event of an impending attack of herbivores and necrotrophs. In response to this host defense mechanism, it is plausible that *P. syringae* might have evolved an additional, COR-independent method of inducing JA signaling. Normally, this mechanism could be masked by the action of GCN2 while it would become de-repressed and active in the *atgcn2* mutant. If such a mechanism were to exist, it would be likely short-lived, and occur during very early stages of infection, as evidenced by the trends of our expression data (Figs. 3d-f). The enhanced expression of MYC2 in the *Pst* DC3118-treated *atgcn2* wears off over time, reaching the levels observed in *Pst* DC3000-treated L*er* at 4 hr time point (Fig. 3d). Similarly, the ANAC019 and ANAC055 expression levels decline in the *atgcn2* plants after a transient induction at the 2 hr time point (Figs. 3e and 3f). However, these transcript levels remain consistently higher than expression observed in the *Pst* DC3118-treated L*er.*

This transient early mis-regulation of MYC2 and NACs could be, in part, responsible for the fact that the *atgcn2* plants spray-inoculated with *Pst* DC3118 no longer show enhanced disease resistance, and have bacterial loads comparable to those of L*er* (Fig. 3b and Supplementary Fig. 2d).

In line with our findings, another node of convergence between ABA and JA signaling was recently reported[6]. HAI1 PP2CA was shown to be induced by ABA through the AREB/ABF transcription factors as well as by *Pst* DC3000 through COR-mediated activation of MYC2. In turn, HAI1 dephosphorylates MPK3 and MPK6 and is necessary for COR-mediated suppression of MPK3/MPK6 activation and immunity. Taken together and consistent with the previous reports, we propose that AtGCN2 is a positive regulator of ABA biosynthesis and signaling, contributes to MYC2-mediated JA signaling, and virulent pathogens potentially target AtGCN2 to establish disease susceptibility.

Supplementary References

1 Ueda, M. *et al.* Noncanonical Function of a Small-Molecular Virulence Factor Coronatine against Plant Immunity: An In Vivo Raman Imaging Approach. *ACS central science* **3**, 462-472, doi:10.1021/acscentsci.7b00099 (2017).

2 Cui, H. *et al.* Antagonism of transcription factor MYC2 by EDS1/PAD4 complexes bolsters salicylic acid defense in Arabidopsis effector-triggered immunity. *Mol Plant*, doi:10.1016/j.molp.2018.05.007 (2018).

3 Liu, L. *et al.* Salicylic acid receptors activate jasmonic acid signalling through a non-canonical pathway to promote effector-triggered immunity. *Nat Commun* **7**, 13099, doi:10.1038/ncomms13099 (2016).

4 Geng, X., Cheng, J., Gangadharan, A. & Mackey, D. The coronatine toxin of Pseudomonas syringae is a multifunctional suppressor of Arabidopsis defense. *Plant Cell* **24**, 4763-4774, doi:10.1105/tpc.112.105312 (2012).

5 Takahashi, F. *et al.* The mitogen-activated protein kinase cascade MKK3-MPK6 is an important part of the jasmonate signal transduction pathway in Arabidopsis. *Plant Cell* **19**, 805-818, doi:10.1105/tpc.106.046581 (2007).

6 Mine, A. *et al.* Pathogen exploitation of an abscisic acid- and jasmonate-inducible MAPK phosphatase and its interception by Arabidopsis immunity. *Proc Natl Acad Sci U S A* **114**, 7456-7461, doi:10.1073/pnas.1702613114 (2017).
