# Supplementary material for: Arabidopsis GCN2 kinase contributes to ABA homeostasis and stomatal immunity
Source: Commun Biol. 2019 Aug 8;2:302. doi: 10.1038/s42003-019-0544-x (PMC6687712; doi:10.1038/s42003-019-0544-x)
Supplement: Supplementary file 2 — Description of Additional Supplementary Files [file 42003_2019_544_MOESM2_ESM.docx]

**Description of Additional Supplementary Files**

**File Name**: Supplementary Data

**Description**:  Excel file with numerical datasets used for main figures.
