# Supplementary material for: Arabidopsis GCN2 kinase contributes to ABA homeostasis and stomatal immunity
Source: Commun Biol. 2019 Aug 8;2:302. doi: 10.1038/s42003-019-0544-x (PMC6687712; doi:10.1038/s42003-019-0544-x)
Supplement: Supplementary file 4 — Reporting Summary [file 42003_2019_544_MOESM4_ESM.pdf]

nature research

Corresponding author(s): Karolina Pajerowska-Mukhtar

Last updated by author(s): Jun 27, 2019

Reporting Summary

Nature Research wishes to improve the reproducibility of the work that we publish. This form provides structure for consistency and transparency in reporting. For further information on Nature Research policies, see Authors & Referees and the Editorial Policy Checklist.

Statistics

For all statistical analyses, confirm that the following items are present in the figure legend, table legend, main text, or Methods section.

n/a	Confirmed	
☐	☒	The exact sample size (n) for each experimental group/condition, given as a discrete number and unit of measurement
☐	☒	A statement on whether measurements were taken from distinct samples or whether the same sample was measured repeatedly
☐	☒	The statistical test(s) used AND whether they are one- or two-sided<br>*Only common tests should be described solely by name; describe more complex techniques in the Methods section.*
☐	☒	A description of all covariates tested
☐	☒	A description of any assumptions or corrections, such as tests of normality and adjustment for multiple comparisons
☐	☒	A full description of the statistical parameters including central tendency (e.g. means) or other basic estimates (e.g. regression coefficient) AND variation (e.g. standard deviation) or associated estimates of uncertainty (e.g. confidence intervals)
☐	☒	For null hypothesis testing, the test statistic (e.g. F, t, r) with confidence intervals, effect sizes, degrees of freedom and P value noted<br>*Give P values as exact values whenever suitable.*
☒	☐	For Bayesian analysis, information on the choice of priors and Markov chain Monte Carlo settings
☒	☐	For hierarchical and complex designs, identification of the appropriate level for tests and full reporting of outcomes
☒	☐	Estimates of effect sizes (e.g. Cohen's d, Pearson's r), indicating how they were calculated

Our web collection on statistics for biologists contains articles on many of the points above.

Software and code

Policy information about availability of computer code

Data collection	Microsoft Excel
Data analysis	Microsoft Excel, GraphPad Prism 8

For manuscripts utilizing custom algorithms or software that are central to the research but not yet described in published literature, software must be made available to editors/reviewers. We strongly encourage code deposition in a community repository (e.g. GitHub). See the Nature Research guidelines for submitting code & software for further information.

Data

Policy information about availability of data

All manuscripts must include a data availability statement. This statement should provide the following information, where applicable:

- Accession codes, unique identifiers, or web links for publicly available datasets
- A list of figures that have associated raw data
- A description of any restrictions on data availability

The tbf1 microarray data have been published previously and are available in the Gene Expression Omnibus (GEO) database under the accession number GSE34047. All other relevant data are available in the manuscript, Supplementary Information files, and from the corresponding author upon request.

Field-specific reporting

Please select the one below that is the best fit for your research. If you are not sure, read the appropriate sections before making your selection.

☒ Life sciences ☐ Behavioural & social sciences ☐ Ecological, evolutionary & environmental sciences

Life sciences study design

All studies must disclose on these points even when the disclosure is negative.

Sample size	We examined sufficient number of samples to reach statistical conclusion according to the specific method utilized in that experiment. The minimal sample size was six.
Data exclusions	No data were excluded.
Replication	All experiments were reproduced to reliably support conclusions stated in the manuscript. In all cases, at least three independent biological replications were performed. The results were reproducible when the plants were not stressed before the experiment.
Randomization	We pick samples randomly to avoid any artificial preferences.
Blinding	We preformed an unbiased analysis to all datasets.

Reporting for specific materials, systems and methods

We require information from authors about some types of materials, experimental systems and methods used in many studies. Here, indicate whether each material, system or method listed is relevant to your study. If you are not sure if a list item applies to your research, read the appropriate section before selecting a response.

Materials & experimental systems

n/a	Involved in the study	
☐	☒	Antibodies
☒	☐	Eukaryotic cell lines
☒	☐	Palaeontology
☒	☐	Animals and other organisms
☒	☐	Human research participants
☒	☐	Clinical data

Methods

n/a	Involved in the study	
☒	☐	ChIP-seq
☒	☐	Flow cytometry
☒	☐	MRI-based neuroimaging

Antibodies

Antibodies used	(1) Anti-EIF2S1 (phospho S51) antibody [E90] (commercial, Abcam) and (2) polyclonal anti-eIF2α antibody custom raised against peptide (IRRRMTPQPMKIRAD) (GenScript)
Validation	(1) Lageix et al., 2008 validated that this antibody specifically recognize the phosphorylation of eIF2a in Arabidopsis. (2) Antibody previpusly described in Zhang Y, et al. (2008) GCN2-dependent phosphorylation of eukaryotic translation initiation factor-2α in Arabidopsis. Journal of Experimental Botany 59(11):3131-3141. However, we raised our own antibody against the epitope described in the above paper.
